# Strategic Brazilian Minerals Applied in the Photocatalytic Ozonation of Rhodamine B Using a Green Lithium Niobate Nanocatalyst Supported on Silica: Kinetic, Thermodynamic, Mechanism, Machine Learning, and Ecotoxicity Study

**DOI:** 10.1021/acsomega.6c00300

**Published:** 2026-03-12

**Authors:** Matheus Londero da Costa, Cristiane dos Santos, Yolice Patricia MorenoRuiz, Giovani Pavoski, Jorge Alberto Soares Tenório, Denise Crocce Romano Espinosa, Jivago Schumacher de Oliveira

**Affiliations:** † Applied Nanomaterials Research Group (GPNAp), Franciscan University (UFN), Santa Maria, Rio Grande do Sul 97010-030, Brazil; ‡ Institute of Chemistry, Federal University of Rio Grande Do Sul (UFRGS), Porto Alegre, Rio Grande do Sul 90040-060, Brazil; § Department of Fundamental Chemistry (DQF), Federal University of Pernambuco (UFPE), Recife, Pernambuco 50670-901, Brazil; ∥ Strategic Technologies Center of Northeast (CETENE), Recife, Pernambuco 50740-545, Brazil; ⊥ Polytechnical School of Chemical Engineering, 28133 University of the Sao Paulo (USP), São Paulo, São Paulo 05508-220, Brazil

## Abstract

This study presents the synthesis and use of a novel heterogeneous lithium niobate nanocatalyst supported on silica (SiO_2_/LiNbO_3_) obtained from rice husk for the degradation of the polluting dye Rhodamine B (RhB) through photocatalytic ozonation. The main objective was to create an eco-friendly nanocatalyst from agro-industrial waste (such as rice husk and lemon) and strategic Brazilian minerals (Nb and Li), fostering a circular economy. Rice husk was used for SiO_2_ extraction, while LiNbO_3_ nanoparticles were biosynthesized by using the hydrothermal method, employing lemon peel extract (*Citrus latifolia*). Characterization confirmed the porous morphology and the creation of nanoparticles (34 nm) with a high surface area, ideal for dye diffusion. Using central composite rotatable design (CCRD), the SiO_2_/LiNbO_3_ system showed high photodegradation efficiency (>97%) in less than 60 min, adhering to the Langmuir–Hinshelwood kinetic model. Superoxide radicals (O_2_^•–^) and valence holes (h+) were the main agents responsible for the degradation process. The ecotoxicity of the final material was low. Furthermore, the use of machine learning (ML) to anticipate the formation of degradation intermediates highlighted the predictive ability of the K-nearest-neighbor (KNN) model.

## Graphical abstract

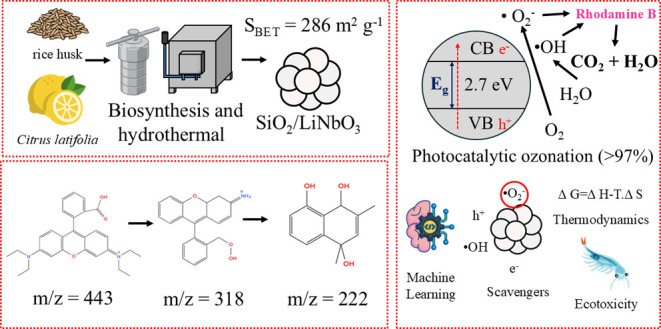

## 1 Introduction

Currently, the agro-industrial sector is one of the largest generators of waste due to agricultural and food processing activities, producing waste such as bagasse,^1^ straw,^2^ husks,^3^ and others. These lignocellulosic materials are high in carbon, oxygen, and silica (SiO_2_).^4^ Due to their high silica content, they have low biodegradability, which can make the waste an environmental liability.^5^ However, the reuse of these materials is gaining prominence as a sustainable and eco-friendly alternative (for example, (e.g.) biofuels).^6^

Rice husk stands out for having a high SiO_2_ content of 15–25% of the total weight.^7^ It is estimated that during rice grain processing, approximately 20% of the mass is generated as husks,^8^ which becomes an environmental challenge. Brazil is one of the world’s largest rice producers, producing over 11 million tons annually, which generates approximately 2.5 million tons of rice husks, posing an environmental problem.^9^ Some studies propose reusing this material for products such as ceramics,^10^ adsorbents,^11^ and composites^12^ to eliminate this waste. Thus, adding value to a waste product promotes a circular economy.

The scarcity of potable water is one of the greatest environmental and social challenges of the 21st century, affecting more than 2 billion people worldwide.^13^ Population growth, coupled with uncontrolled urbanization and industrial activities, intensifies the consumption and contamination of water resources.^14^ It is estimated that by 2050, approximately 50% of the world’s population may live in areas with various water deficiencies,^15^ highlighting the urgency of sustainable strategies for the preservation of water resources.

Rhodamine B (RhB) is a xanthene dye widely used in textiles and cosmetics and as a fluorescent marker in bioassays.^16^ However, due to its high chemical stability and potential toxicity, its presence in industrial effluents represents a serious environmental risk, potentially causing mutagenic effects and disturbances in aquatic ecosystems.^17^ Furthermore, conventional water treatment techniques frequently fail to completely remove this pollutant, generating persistent byproducts.^18^

Adsorption is the process by which chemical species accumulate on the surface of a solid adsorbent due to physical or chemical interactions between the contaminant and the material. This process is affected by elements such as surface area, porosity, chemical composition of the surface, pH, and adsorbate concentration, being particularly efficient for the rapid removal of organic and inorganic contaminants from the liquid phase. However, by transferring the contaminant to the adsorbent, it ends up creating a new environmental liability.^19^ On the other hand, photocatalysis is based on the activation of a semiconductor material by means of light, which generates electron–hole pairs that can induce chemical reactions on the catalyst surface. These reactions result in the transformation or degradation of contaminants, generally converting them into less toxic or mineralized species, characterizing it as a sustainable process by employing light as an energy source.^20,21^ In this scenario, advanced oxidation processes (AOPs) emerge as efficient alternatives, combining powerful oxidants such as ozone (O_3_), catalysts, and light irradiation for the degradation of organic compounds.^22^ Heterogeneous photocatalysis has been extensively investigated due to the ability of metallic semiconductors to generate reactive oxygen species (ROS) under UV/visible light, promoting the breakdown of organic molecules.^23^ When associated with O_3_, which acts as a strong oxidizing agent, the efficiency of the process can be significantly amplified due to the synergistic formation of different radicals generated in the middle such as O_2_^•–^ which act in the mineralization of the target pollutants.^24^

In Brazil, during the fifth National Conference on Science, Technology and Innovation (5CNCTI) held in 2024, strategic Brazilian minerals for technology development were defined, highlighting niobium (Nb), lithium (Li), and silicon (Si).^25,26^ Thus, there is potential for the development of national technologies around these elements.

Supported catalysts consist of photoactive materials dispersed on an inert matrix such as SiO_2_ or carbon, which increases the surface area and stability of the catalyst, optimizing its efficiency in heterogeneous chemical reactions;^27^ typically, 5% of the photoactive phase is used for the matrix phase.^28^ When these systems have nanometric dimensions, they are called nanocatalysts, characterized by high reactivity, selectivity, and many available active sites.^29^ Green synthesis seeks to produce these materials using environmentally sustainable methods, employing nontoxic solvents, moderate temperatures, and reagents of natural origin, reducing environmental impacts and promoting cleaner and more efficient chemical processes.^30^

The objective of this work is to biosynthesize lithium niobate (LiNbO_3_) nanoparticles using lemon extract (*Citrus latifolia*) via a hydrothermal route and support them on SiO_2_ extracted from rice husks, evaluating the degradation of the RhB dye by photocatalytic ozonation through kinetic, thermodynamic, ecotoxicity, and photodegradation mechanism studies using machine learning. The objective is to evaluate the possibility of creating an environmentally friendly photocatalytic material from waste, in conjunction with strategic Brazilian minerals, generating added value to a national technology, given that LiNbO_3_ has scarce studies in the literature highlighting its use in green synthesis.

## 2 Results and Discussion

### 2.1 Characterization

Figure 1 shows the FTIR-ATR spectrum of SiO_2_, LiNbO_3_, and SiO_2_/LiNbO_3_. For Figure 1, we can observe the peaks at 1050 cm^–1^, 801 cm^–1^, and 459 cm^–1^ were attributed to Si–O stretching vibration, Si–O–Si symmetric elongation, and Si–O–Si bending, respectively,^31^ which confirms the synthesis of SiO_2_. While for LiNbO_3_, the niobate groups are noted, which exhibited two bands at 860 cm^–1^ and 585 cm^–1^ due to Nb=O and Nb–O–Nb stretching vibrations. The band at 477 cm^–1^ is attributed to specific Li–O bond vibrations^32^ to SiO_2_/LiNbO_3_; there is an overlap of the material stretches.

**Figure 1:**
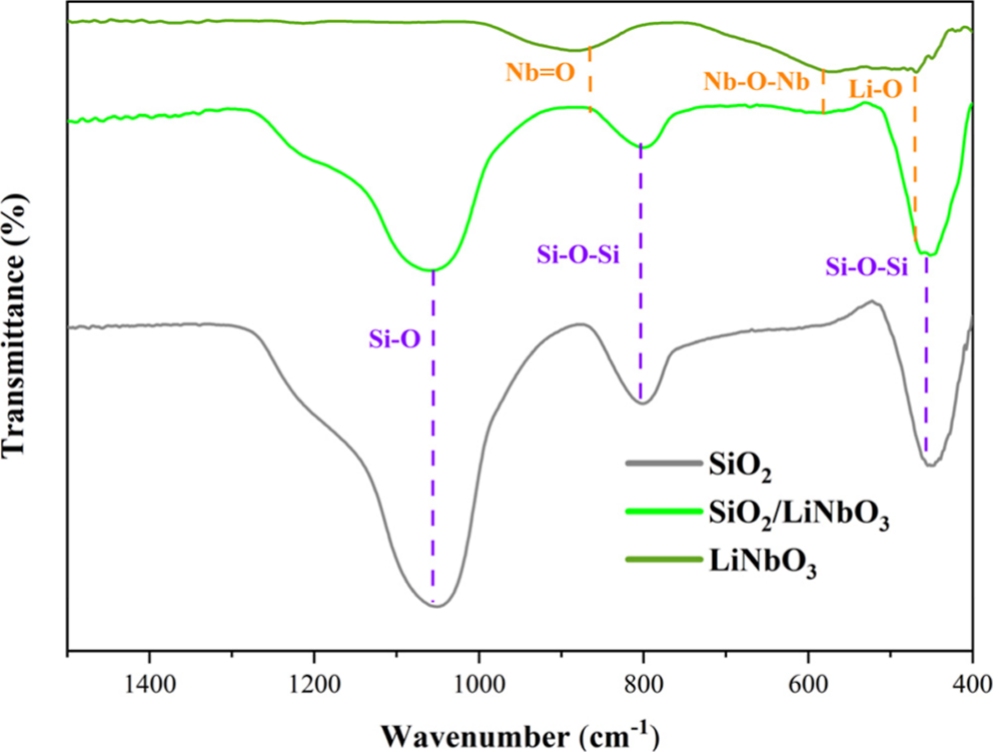
ATR-FTIR spectrum obtained from SiO_2_, SiO_2_/LiNbO_3_, and LiNbO_3_.

Figure 2 presents the SEM-FEG micrographs and EDX results for the catalytic support (SiO_2_), photoactive phase (LiNbO_3_), and supported nanocatalyst (SiO_2_/LiNbO_3_), where it was possible to identify a heterogeneous morphology and porous (Figure 2a,c,e) characteristic of a supported nanocatalyst ideal for promoting the intraparticle diffusion of RhB dye molecules. Regarding the EDX results, it was possible to verify the majority elemental composition of Si, Nb, and O, confirming the effectiveness of the synthesis process of the samples SiO_2_ (Si 40.2 wt % and O 30.7 wt %) (Figure 2b), LiNbO_3_ (Nb 69.4 wt % and O 19.1 wt %) (Figure 2e), and SiO_2_/LiNbO_3_ (O 45.1 wt %, Si 30.3 wt %, and Nb 0.6 wt %) (Figure 2h), confirming the proportion of Nb under SiO_2_ determined in the impregnation process. It was also possible to note the presence of nanoparticles (<100 nm)^33^ onto the surface of all materials as shown in Figure 2c,f,i. indicating an average size of 41.06 ± 15.13 nm, 31.77 ± 13.82 nm, and 34.25 ± 10.71 nm to SiO_2_, LiNbO_3_, and SiO_2_/LiNbO_3_, respectively. The sizes are close to those found in the literature, 20 nm for SiO_2_ ^34^ and LiNbO_3_.^35^

**Figure 2:**
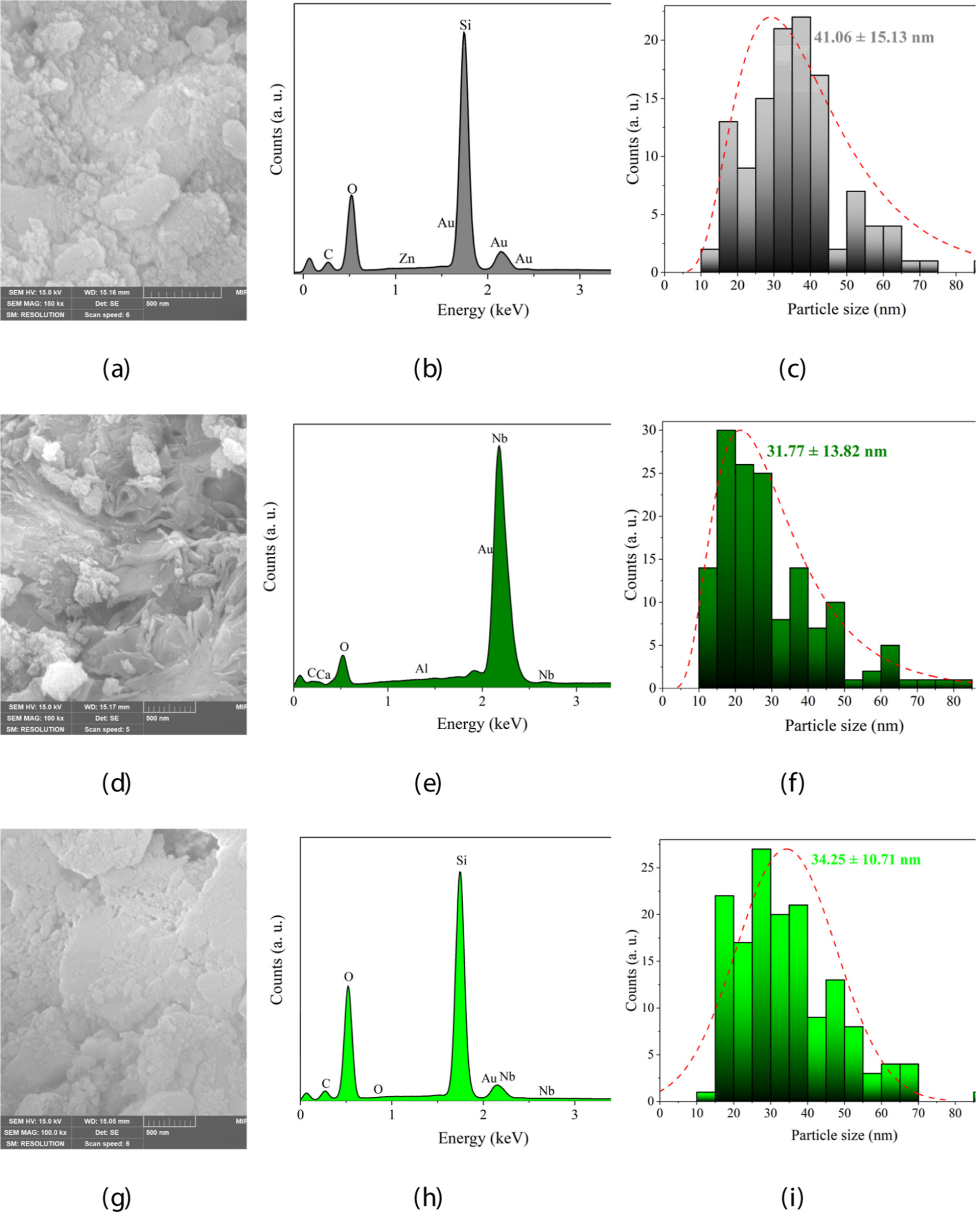
SEM micrographs of (a) SiO_2_, (d) LiNbO_3_, and (g) SiO_2_/LiNbO_3_; EDX results of (b) SiO_2_, (e) LiNbO_3_, and (h) SiO_2_/LiNbO_3_; and mean particle size distribution of (c) SiO_2_, (f) LiNbO_3_, and (i) SiO_2_/LiNbO_3_.

Figure 3 shows the N_2_ adsorption isotherms. Figure 3 shows that all samples presented a type IV­(a) isotherm with H3 hysteresis, indicating a mesoporous material (2 to 50 nm) with slit-type pores and/or aggregated plates with a lamellar structure, as can be observed in Figure 3.^36^ Figure S1 shows the DRS spectrum. Table 1 presents textural, structural, and optical properties. In Table 1, the synthesized SiO_2_ showed a higher *S*_BET_ than that reported in the literature (320 > 182 m^2^ g^–1^), while *V*_p_ and *D*_p_ were lower.^37^ For the synthesized LiNbO_3_, a significantly higher BET was shown compared to another study (259 > 4 m^2^ g^–1^), indicating that with the increasing crystallinity of the material, there is a loss of surface area.^38^ With the addition of LiNbO_3_ under SiO_2_, there was a significant increase in *S*_BET_, while *V*_p_ and *D*_p_ decreased due to possible pore obstruction. While the DRS analysis showed an *E*_g_ close to 3 eV for SiO_2_ and LiNbO_3_, after the supported material test, this value decreased to 2.75 eV, which is expected for supported materials. The green LiNbO_3_ sintered in this work showed a lower eg compared to the literature (3.77 eV); this is due to the low calcination temperature (450 °C), which, because it does not have high crystallinity, results in a higher surface area compared to other metallic oxides.^39^ As can be seen in the XRD analysis, all samples showed a semiamorphous shape, as shown in Figure S3, due to the low calcination temperature used. For the SiO_2_ sample, the typical profile of a predominantly amorphous material is observed, characterized by a broad band centered approximately between 20 and 25° (2θ). This diffuse halo is widely reported for amorphous silica and is associated with the absence of long-range order. The lack of narrow and well-defined peaks confirms the low crystallinity index, suggesting that the structure is essentially disordered.^40^ In the diffractogram of LiNbO_3_, although areas of higher intensity are observed in positions corresponding to the typical reflections of the material, the peaks exhibit considerable broadening and reduced definition. The regions near 23°, 32–35°, and 39° may be related to the (012), (104)/(110), and (202) planes, respectively, which correspond to the most pronounced reflections of the rhombohedral phase (*R*3*c*).^41^ However, the broadening and overlap suggest that the material has a limited crystallinity. This behavior can be explained by factors such as the small size of the crystallites, the existence of structural defects, microdeformations in the lattice, or residual contribution of partially amorphous phases. Thus, the crystallinity index can be classified as moderate, below what is expected for a highly crystalline LiNbO_3_, but adequate to indicate the formation of the desired phase. In the SiO_2_/LiNbO_3_ composite, the predominant presence of an amorphous halo is again noted, analogous to that observed in pure silica but with subtle alterations in the shape and intensity of the diffuse band. The lack of well-defined LiNbO_3_ peaks indicates that the crystalline particles are highly dispersed, possibly due to a further reduction in crystallite size or partial encapsulation by the SiO_2_ matrix. This phenomenon is frequent in supported materials, where the active phase has a low volume fraction or high nanometric dispersion, leading to attenuated diffractometry signals.^42^ The profile suggests that the composite has a low overall crystallinity index, being predominantly amorphous due to the silica. However, the structural presence of LiNbO_3_ can be inferred indirectly through small alterations in the halo.

**Figure 3:**
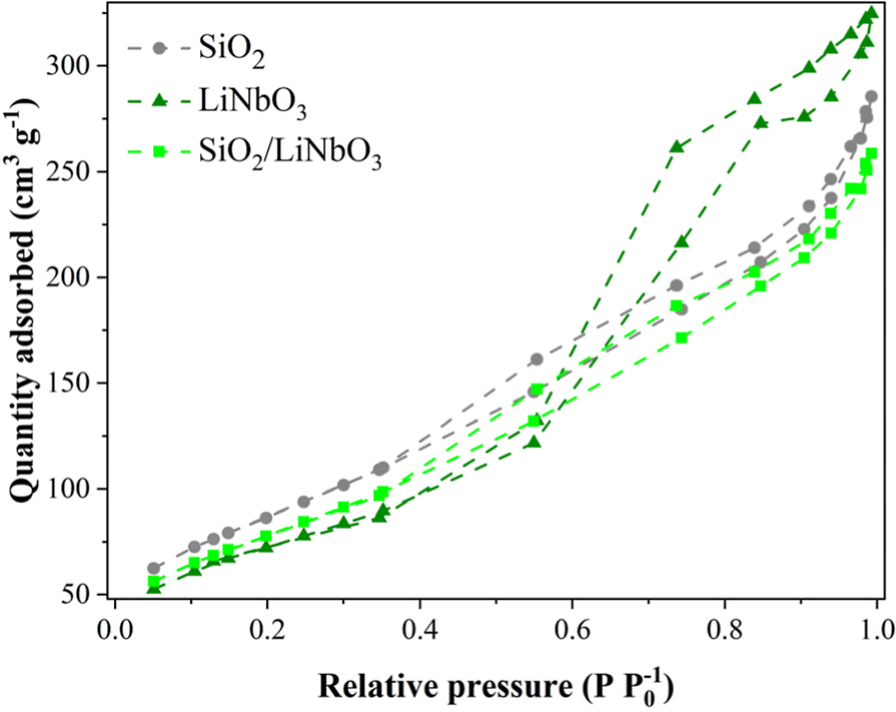
N_2_ adsorption isotherm of SiO_2_, LiNbO_3_, and SiO_2_/LiNbO_3_.

**Table 1:** Textural, Structural, and Optical Properties of SiO_2_, LiNbO_3_, and SiO_2_/LiNbO_3_

sample	S_BET_ (m^2^ g^–1^)	*V*_p_ (cm^3^ g^–1^)	*D*_p_ (nm)	*E*_g_ (eV)
SiO_2_	320.0	0.43	5.4	3.03
LiNbO_3_	259.9	0.50	6.6	3.06
SiO_2_/LiNbO_3_	286.9	0.39	5.3	2.75

Figure 4 shows the results obtained in the pH_ZCP_ test. We can see that SiO_2_ has a pH_ZCP_ close to neutral (7.23), while LiNbO_3_ has acidic characteristics (5.13). When added to SiO_2_, it makes the surface slightly more acidic; thus, the supported nanocatalyst (SiO_2_/LiNbO_3_) obtained a pH_ZCP_ of 5.46. Since RhB is a cationic dye that prefers dehydrogenase surfaces (pH > pH_ZCP_) to adsorb onto the material’s surface, this study used a natural pH of the RhB solution (≈6), favoring adsorption at the active sites of the nanocatalyst without the addition of pH regulators.^43^

**Figure 4:**
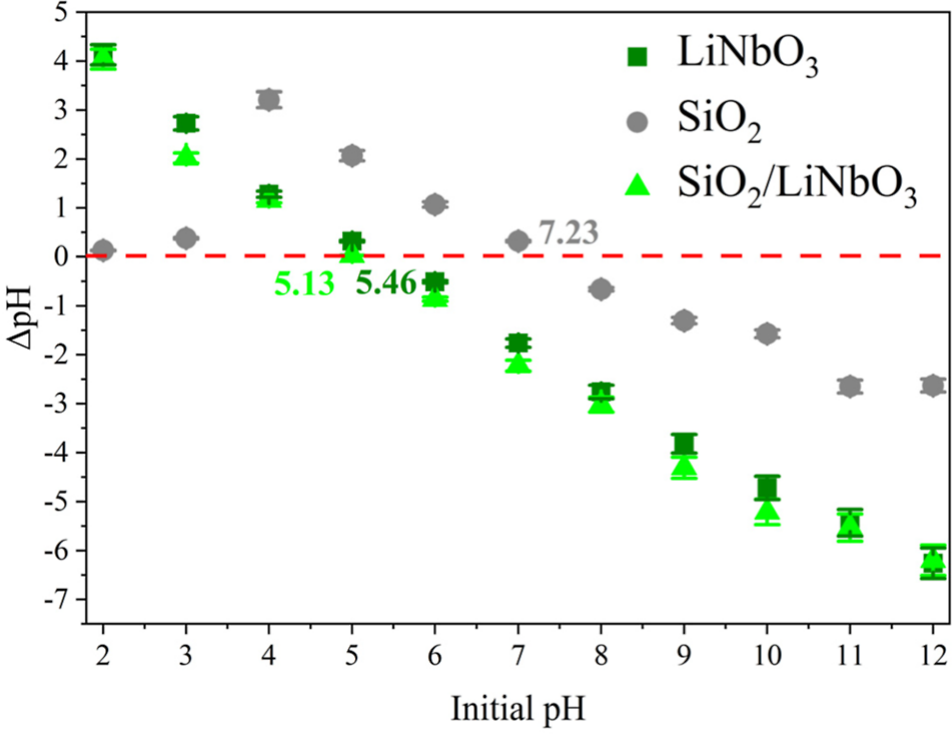
pH_ZCP_ of SiO_2_, LiNbO_3_, and SiO_2_/LiNbO_3_.

### 2.2 Central Composite Rotatable Design

Figure 5a shows the evaluation of RhB removal, where catalyst concentrations in lower and higher ranges showed greater removal when using lower RhB concentrations. This is possibly due to the catalyst tending to agglomerate and form clusters, decreasing the contact area with the dye.^44^ The ideal point was achieved using 0.2 (g L^–1^) SiO_2_/LiNbO_3_ and 10 (mg L^–1^) RhB, where a removal of 98.18% and a photodegradation of 97.26% of RhB were obtained. In Figure 5b, it is possible to observe that the apparent kinetic rate (*k*_1_) tends to increase when less catalyst and RhB are used, also justifying the hypothesis of catalyst agglomeration. Under ideal conditions, a *k*_1_ of 0.094 (min^–1^) was obtained. Figure 6c shows the SHAP graph obtained by evaluating the photocatalytic ozonation of RhB, where it is possible to verify that the variables with the greatest significance for the process are the reaction time (min) and the O_3_ flow rate (mg h^–1^), which have a positive effect, while the RhB concentration has a negative effect. Finally, the catalyst concentration has an intermediate positive significance in the photodegradation.

**Figure 5:**
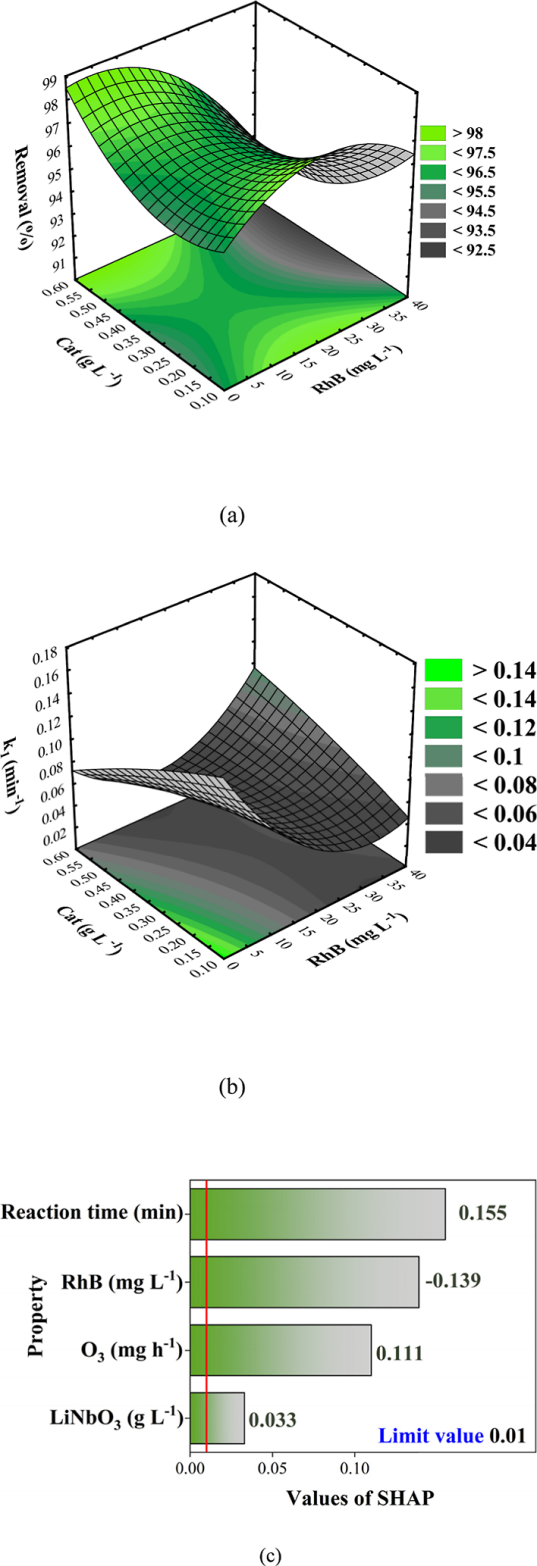
CCRD concentration of LiNbO_3_ (Cat (g L^–1^)) versus concentration of RhB (mg L^–1^) evaluating (a) removal (%), (b) *k*_1_ (min^–1^), and (c) SHAP graph.

**Figure 6:**
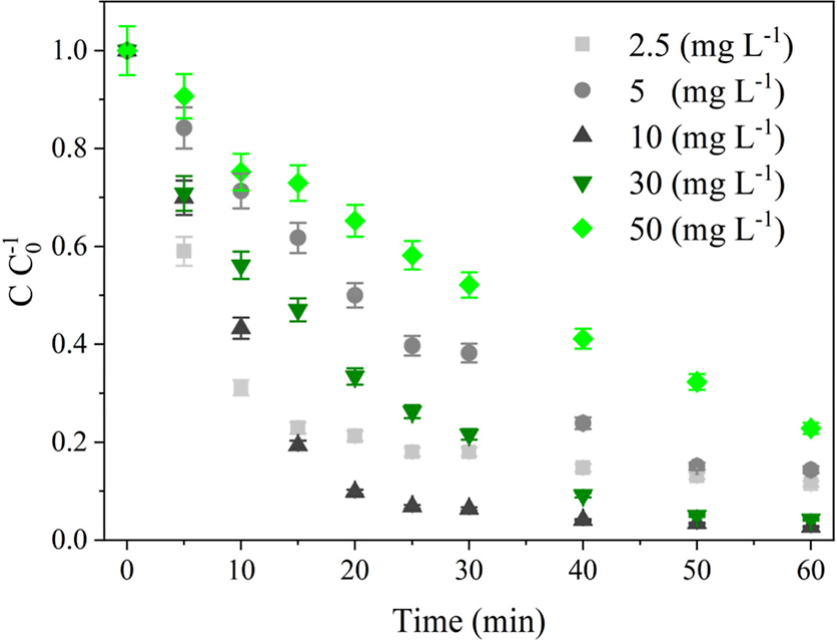
Evaluation of RhB photodegradation at different concentrations using 0.2 (g L^–1^) of SiO_2_/LiNbO_3_ and 375 (mg h^–1^) of O_3_ flow.

### 2.3 Kinetics

Figure 6 presents the kinetic study of RhB removal using 0.2 (g L^–1^) of SiO_2_/LiNbO_3_, where the RhB concentration varied from 2.5 to 50 (mg L^–1^) using 375 (mg h^–1^) O_3_ flow rate. Table 2 shows the results obtained from the kinetic study. The results indicate that the effectiveness in removing RhB varies considerably according to the initial dye concentration. Degradation ranged from 77.16% to 97.27%, with the highest value recorded for the intermediate concentration of 10 (mg·L^–1^). This behavior demonstrates the presence of an ideal concentration point, where there is a balance between the amount of molecules adsorbed and the generation of reactive species in the photocatalytic system; this trend is confirmed by the values *k*_1_.^45^ The highest constant was again recorded for 10 mg·L^–1^ (0.094 min^–1^), indicating that, in this scenario, the system offers greater accessibility to the active sites and greater effectiveness in the interaction between O_3_, photogenerated species, and the dye. At lower concentrations (2.5 mg·L^–1^), although the final degradation is greater, the value of *k*_1_ is reduced, indicating a limitation due to the lower availability of molecules for the reaction. At higher concentrations (30 and 50 mg·L^–1^), a decrease in *k*_1_ is observed, which is consistent with the saturation of catalytic sites, intensification of competition for radicals, and a possible shadowing effect, which reduces the effectiveness of the photogeneration of oxidizing species.^46^

**Table 2:** Parameters Obtained in the Kinetic Study of RhB Photodegradation at Different Concentrations Using 0.2 (g L^–1^) of SiO_2_/LiNbO_3_ and 375 (mg h^–1^) of O_3_ Flow

	RhB concentration (mg L^–1^)				
	2.5	5	10	30	50
photodegradation (%)	88.52	85.66	97.27	95.80	77.16
*k*_1_ (min^‑1^)	0.087	0.035	0.094	0.055	0.023
*t*_1/2_ (min)	7.91	20.09	7.40	12.62	30.79
*R* ^2^	0.895	0.996	0.985	0.994	0.993

Analysis of *t*_1/2_ aids this interpretation: the shortest *t*_1/2_ (7.40 min) for 10 (mg·L^–1^) indicates a more accelerated degradation, while the longest *t*_1/2_ occurs at 50 (mg·L^–1^) (30.79 min), highlighting the restriction imposed by high dye concentrations. The *R* ^2^ values, all above 0.89, suggest that the fit to the pseudo-first-order L–H kinetic model is adequate for all analyzed conditions. This confirms that the process is mainly controlled by mechanisms that depend on the concentration of the adsorbed substrate and the presence of reactive species on the catalytic surface.^47^

### 2.4 Comparative Evaluation of Catalytic Systems

Figure 7 presents the evaluation of the catalytic systems alone and in combination. We can see that O_3_ alone cannot completely degrade RhB. For the systems with O_3_ + LiNbO_3_ and with O_3_ + SiO_2_, both reach maximum degradation around 40 min and remain constant. Finally, the combined materials of O_3_ + SiO_2_/LiNbO_3_ reach maximum degradation in 25 min, varying little after that time, showing the synergistic efficiency of the combined materials, which act at a faster rate in the photodegradation of RhB.

**Figure 7:**
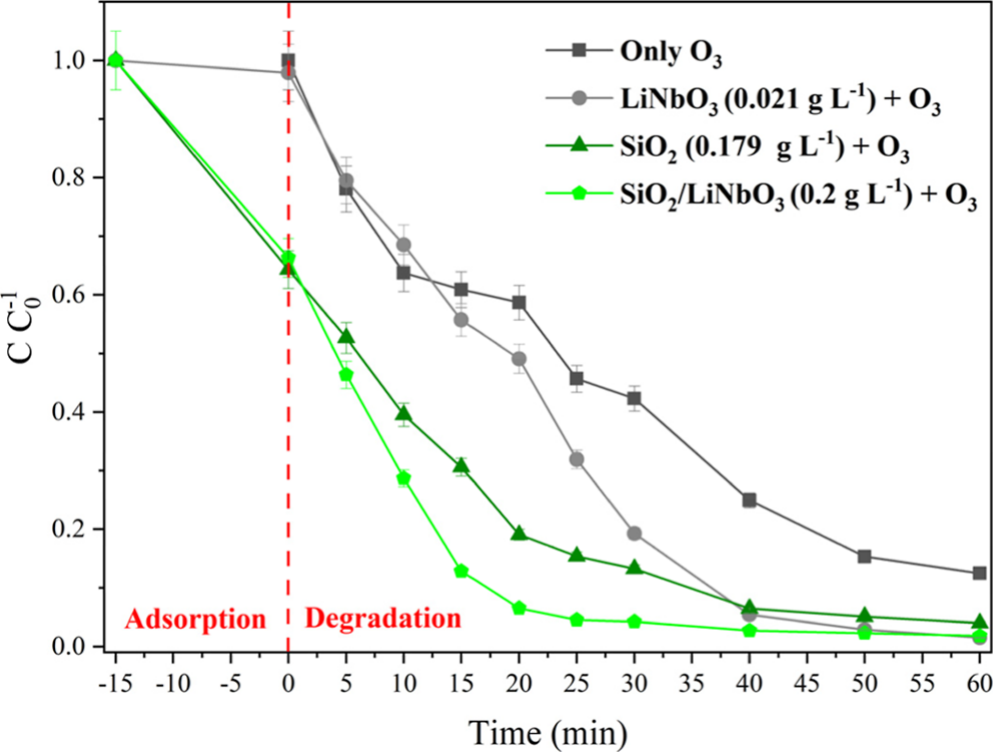
Comparison of separate and combined catalytic systems using 375 mg h^–1^ of O_3_ and 10 mg L^–1^ of RhB.

### 2.5 Thermodynamics

Table 3 presents the thermodynamic parameters obtained where the negative value of the activation enthalpy (Δ^‡^ *H* ^0^ = −41.6 kJ·mol^–1^) indicates that the formation of the transition state is energetically favored, suggesting strong intermediate stabilization resulting from the interaction between the RhB, O_3_-derived species and the catalyst surface.^48^ This behavior is characteristic of associative mechanisms, in which adsorption plays a crucial role in the reaction pathway.^49^ The activation entropy also exhibited a significant negative value (Δ^‡^ *S* ^0^ = −0.393 kJ·(mol K)^−1^), indicating that the activated state has a more ordered structure compared to the free reactants.^50^ In photocatalytic systems, this organization is expected, as the adsorbed molecule needs to be specifically oriented for charge transfer or attack by reactive radicals generated during irradiation to occur.^51^

**Table 3:** Thermodynamic Parameters Obtained Using 0.2 (g L^–1^) of SiO_2_/LiNbO_3_, 10 (mg L^–1^) of RhB, and 375 (mg h^–1^) of O_3_ Flow

	transition theory				Arrhenius theory		
temperature (K)	Δ^‡^ *G* ^0^ (kJ mol^–1^)	Δ^‡^ *H* ^0^ (kJ mol^–1^)	Δ^‡^ *S* ^0^ (kJ (mol K)^−1^)	*R* ^2^	*A*_*e*_ (kJ mol^–1^)	A (min^–1^)	*R* ^2^
298.15	75.6	–41.6	–0.393	0.96	1.6 × 10^–6^	1.003	0.95
308.15	79.5						
318.15	83.5						

The increase in Δ^‡^ *G* ^0^ values with increasing temperature confirms the typical behavior of systems with Δ^‡^ *H* ^0^ < 0 and Δ^‡^ *S* ^0^ < 0, where the process becomes less spontaneous at higher temperatures.^52^ This corroborates the notion that the crucial rate step depends on adsorption and molecular organization, processes favored at lower temperatures.^53^ Taken together, the results suggest that photocatalytic ozonation is constrained by the creation of a rigidly structured and stabilized transition state on the catalyst surface, which is compatible with surface mechanisms based on the L–H model.

The thermodynamic parameters obtained offer important insights into the photocatalytic ozonation mechanism. Note that the Δ^‡^ *G* ^0^ values are positive and increase with increasing temperature, ranging from 75.6 to 83.5 kJ mol^–1^. This behavior suggests that a crucial phase of the rate faces a specific energy barrier, aligned with a process governed by surface reactions, as anticipated by the L–H kinetic model. In systems based on this model, the overall rate depends on the adsorption of reactant species and chemical reactions on the incident surface. In this case, we expect the activation free energy to have positive values, as it reflects the energy required for the formation of the activated complex. The negative value of Δ^‡^ *H* ^0^ (−41.6 kJ mol^–1^) indicates that the creation of the transition state is exothermic. This result is consistent with mechanisms involving highly reactive radical species, whose creation can release energy due to partial stabilization in the activated complex. Mechanically, this behavior can be related to the interaction among O_3_, adsorbed molecules, and photogenerated charge carriers, which facilitates the production of reactive oxygen species on the catalytic surface. The parameter Δ^‡^ *S* ^0^, also negative (−0.393 kJ (mol K)^−1^), suggests a reduction in entropy during the formation of the transition state. This result indicates greater structural organization, characteristic of mechanisms in which there is an association between the adsorbed species on the surface. This interpretation is especially significant within the framework of L–H kinetics since the model considers that the limiting steps involve adsorbed species reacting with each other or with radicals formed at the solid–liquid interface. The reduction in entropy, therefore, reinforces the hypothesis that the transition state involves a more organized arrangement of the reacting species on the catalyst surface. This analysis is complemented by the parameters obtained from the Arrhenius equation. The relatively low activation energy value (*A*_*e*_ ≈ 1.6 × 10^–6^ kJ mol^–1^) suggests that, once adsorption equilibrium is reached, the reaction occurs in an energetically easy manner. This behavior is commonly seen in radical-mediated processes, where the chemical step has a lower energy dependence than that in exclusively thermal systems. A high correlation coefficient (*R* ^2^ ≈ 0.95) indicates that the kinetic model used fits well, showing that the experimental data and the mathematical description of the process are consistent.

### 2.6 Effect of Scavengers on Heterogeneous Photocatalysis

Figure 8 shows the effect of different scavengers applied in photocatalytic ozonation. We can observe that the condition without scavengers showed high efficiency (97.26%), suggesting that in the original system, several oxidizing species act simultaneously in the oxidation of the dye. The inclusion of IA, a scavenger of ^•^OH, led to an efficiency almost equal to that of the control condition (97.03%). This behavior indicates that, even with the presence of ^•^OH in the system, they do not act as the main oxidative pathway or that their suppression is compensated by the combined action of other highly reactive species formed in the presence of O_3_, such as O_3_^•^and O_2_^•–^.^54^ The degradation was reduced to 87.67% due to the action of EDTA as an effective h^+^ scavenger. This reduction suggests that holes play an important role in the oxidation mechanism, possibly initiating the oxidation of RhB and promoting the subsequent formation of secondary radicals. The moderate decrease indicates that h^+^ is involved in the process but does not represent the main pathway. The inclusion of PD, a selective e^–^ scavenger, decreased the level of degradation to 85.15%. This behavior indicates that photoexcited electrons play an important role in the reduction of O_3_ and the formation of derived reactive species, such as O_2_^•–^.^55^ Removing them reduces the rate of conversion of the O_3_ into oxidizing radicals, resulting in slower degradation kinetics. Finally, the use of AA as an O_2_^•–^ scavenger resulted in the most significant reduction in efficiency, reaching only 62.85% photodegradation. This result demonstrates that O_2_^•–^ is the fundamental species in the oxidation process, playing the role of the main intermediate in the degradation of RhB during photocatalytic ozonation.

**Figure 8:**
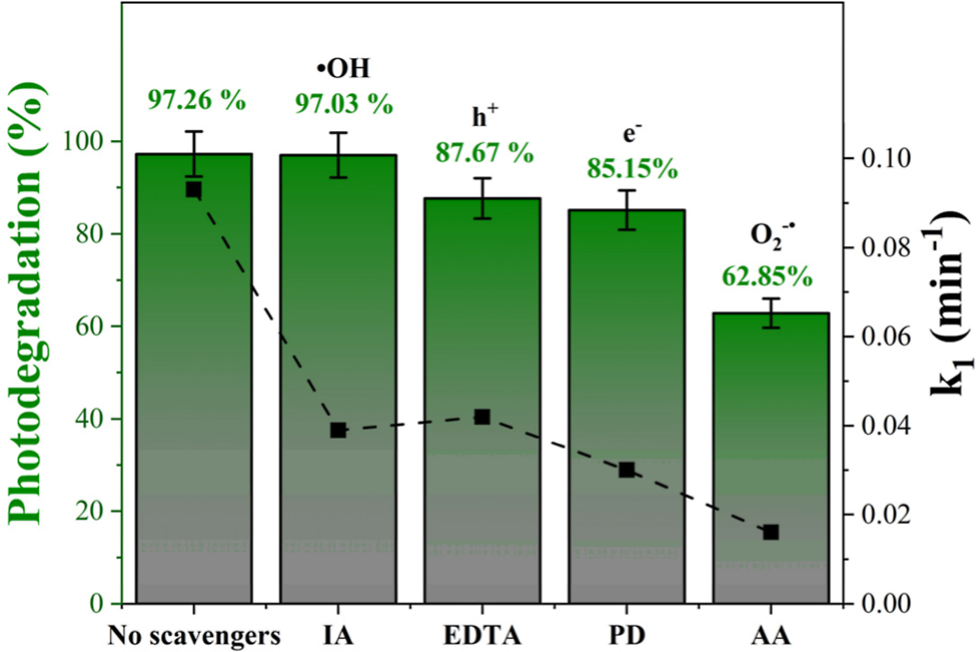
Effect of different scavengers on photocatalytic ozonation using 10 mg L^–1^ of RhB, 0.2 g L^–1^ of LiNbO_3_, and 375 mg h^–1^ of O_3_.

The relationship of these results to the radical mechanism is especially relevant considering that the peptide O_2_^•‑^ was recognized as the most predominant species in the process. The predominance of this reactive species is consistent with the observed thermodynamic parameters. The formation of O_2_^•‑^ results directly from the capture of electrons generated by photosynthesis by dissolved oxygen, a process facilitated in semiconductors that exhibit efficient charge separation, such as LiNbO_3_. The presence of a negative Δ^‡^ *H* ^0^ indicates that the formation and stabilization of these reactive species provide energy for the reaction to proceed, while the negative Δ^‡^ *S* ^0^ demonstrates the associative nature of the interactions between radicals and adsorbed molecules. In the L–H model, the rate-limiting step can be understood as the reaction between the adsorbed pollutant and the radical species generated on the surface. In this context, the radical O_2_^•‑^ plays a fundamental role, both by directly participating in oxidation reactions and by assisting in the formation of other oxidizing species, such as hydroxyl radicals. The dependence of the activation free energy on temperature reinforces the notion that the process is not only diffusional but also controlled by chemical interactions on the surface. The thermodynamic and kinetic parameters suggest that the process is governed by associative surface reactions, with a considerable contribution from radical species. The predominance of the radical oxidizing O_2_^•‑^ as the most influential oxidizing agent is corroborated both by the reduction in entropy in the transition state and by the observed enthalpic behavior. This indicates that the effectiveness of the system is strongly related to the production, stabilization, and reactivity of these species at the catalytic interface.

### 2.7 Reuse Tests

The data shown in Figure 9 demonstrate that the nanocatalyst maintains a high photocatalytic efficiency during multiple reuse cycles. Photodegradation efficiencies remained consistently high, ranging from 94.50% to 98.22% from the first to the fifth use. This behavior suggests that the material maintains good structural and chemical stability under the reaction conditions used, without a significant loss of catalytic activity. The small variation observed between cycles is within the usual experimental fluctuations and can be attributed to factors such as possible transient surface changes. The lack of a consistent trend of reduced efficiency indicates that deactivation phenomena, such as saturation of active sites, leaching of catalytically relevant species, or changes in crystal structures, are insignificant in the analyzed period. The small difference recorded between the cycles is within normal experimental variations and can be explained by elements such as possible temporary surface changes. The absence of a consistent downward trend in efficiency suggests that deactivation phenomena, such as saturation of active sites, leaching of catalytically relevant species, or changes in crystal structures, are not relevant during the period studied. Furthermore, the preservation of catalytic activity after multiple cycles demonstrates the material’s resistance to oxidizing species produced during the ozone photocatalysis process. In such systems, catalyst stability is a crucial factor, as highly reactive radicals can cause surface degradation or undesirable restructuring. The observed performance suggests that the interaction among LiNbO_3_, radiation, and ozone does not significantly affect the active sites responsible for the production and transfer of reactive species. From a practical perspective, the high reusability of the nanocatalyst constitutes a significant benefit for environmental applications, as it helps to lower operating costs and reduces the production of solid waste. Taking together, the results indicate that the material not only exhibits high photocatalytic activity but also adequate durability, fundamental characteristics for its technological application in advanced wastewater treatment processes.

**Figure 9:**
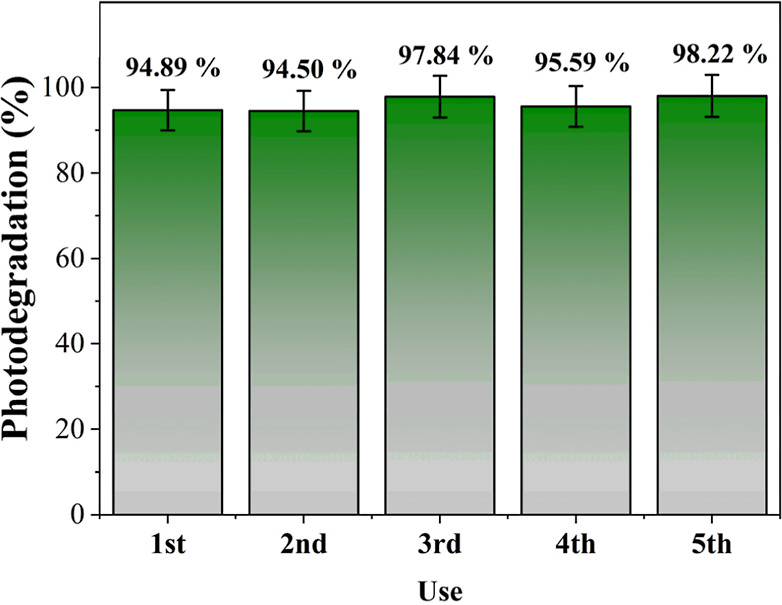
Effect of reusing the nanocatalyst on photocatalytic ozonation using 10 mg L^–1^ of RhB, 0.2 g L^–1^ of LiNbO_3_, and 375 mg h^–1^ of O_3_.

### 2.8 Machine Learning

The use of ML algorithms to predict the *m*/*z* values related to intermediates generated during the photodegradation of RhB showed a solid performance in Table 4. This suggests that the structural patterns originating from the experimental variables are sufficiently informative for the models to identify the connections between the occurrence conditions and the products formed. All analyzed models exhibited high coefficients of determination on the training set (*R* ^2^ ranging from 0.93 to 0.99), demonstrating a remarkable ability to adapt to the particularities of the data set. However, when considering both the *R* ^2^ values and the MAE on the test set, significant differences in the generalization capacity of the algorithms are observed.

**Table 4:** Best Parameters Presented in the ML Study

		train		test	
model	parameters	*R* ^2^	MAE	*R* ^2^	MAE
MLP	hidden layer sizes: (100, 50)	0.933	20.76	0.879	30.19
	alpha: 0.001				
	activation: relu				
random Forest	n estimators: 200	0.966	15.96	0.850	36.29
	max depth: 10				
KNN	weights: distance	0.994	2.85	0.925	22.35
	p: 1				
	n neighbors: 2				
gradient Boosting	n estimators: 100	0.980	12.94	0.892	28.89
	max depth: 3				
	learning rate: 0.1				
extra Trees	n estimators: 200	0.985	8.55	0.913	24.94
	min samples split: 2				
	max depth: 10				

The KNN model demonstrated the best balance between performance and stability, achieving an *R* ^2^ of 0.952 in the test and the lowest MAE = 22.35. This demonstrates a high predictive capacity for *m*/*z*, even with nonlinear and potentially noisy data. This indicates that the local configuration of the data, in other words, the similarity between nearby samples in multivariate space, is a predominant aspect in the process of RhB intermediate formation.^56^ This may reflect the presence of consistent molecular fragmentation patterns linked to the experimental variables.

The Extra Trees Regressor also showed remarkable performance (*R* ^2^ = 0.913; MAE = 24.94), demonstrating that tree ensemble-based techniques are extremely effective for modeling complex chemical systems, as they can capture nonlinear interactions without the need for a pre-established functional form. The Random Forest performed slightly worse, with *R* ^2^ = 0.850 and a higher MAE, indicating that, despite its effectiveness, its generalization ability was limited by the inherent variability of the data set. The Gradient Boosting algorithm showed good performance (*R* ^2^ = 0.892) but with a higher mean error. This indicates that, although it has high fitting power, the sequential boosting process may have amplified the noise in the experimental variables.^57^

The MLP Regressor performed at an intermediate level, indicating that although neural networks are appropriate for modeling highly nonlinear processes, the available data set may not be large enough to fully utilize the neural architecture, leading to greater error variance. In comparison, the overall analysis shows that neighborhood-based and random tree models were more efficient, as they maintain structural relationships and multivariate interactions without requiring large amounts of data.^58^ Thus, the results confirm that machine learning is an effective tool for predicting photodegradation intermediates, enabling a correlation between operational variables and fragmentation pathways.

Figure 10 presents the photodegradation mechanism predicted by the ML KNN model using the conditions of this work: *E*_g_: 2.75 eV, *S*_BET_: 286.9 m^2^ g^–1^, and RhB concentration at time *t* (min) with 100% peak intensity. First, RhB (*m*/*z* 443) was subjected to preferential oxidative attack in areas of higher electron density, particularly in the aminodialkylated groups and the xanthenic structure. This attack causes dealkylation, resulting in species of a lower molecular weight. The first competing species (*m*/*z* 318, 10 min) shows the removal of ethylamino groups and the formation of hydroxylated groups, indicating that oxidizing radicals facilitated both C–N cleavages and oxygen insertions.^59^ The structural alteration considerably decreased electronic conjugation, resulting in the gradual disappearance of the typical color of RhB, which is consistent with the initial chromophore breakdown.

**Figure 10:**
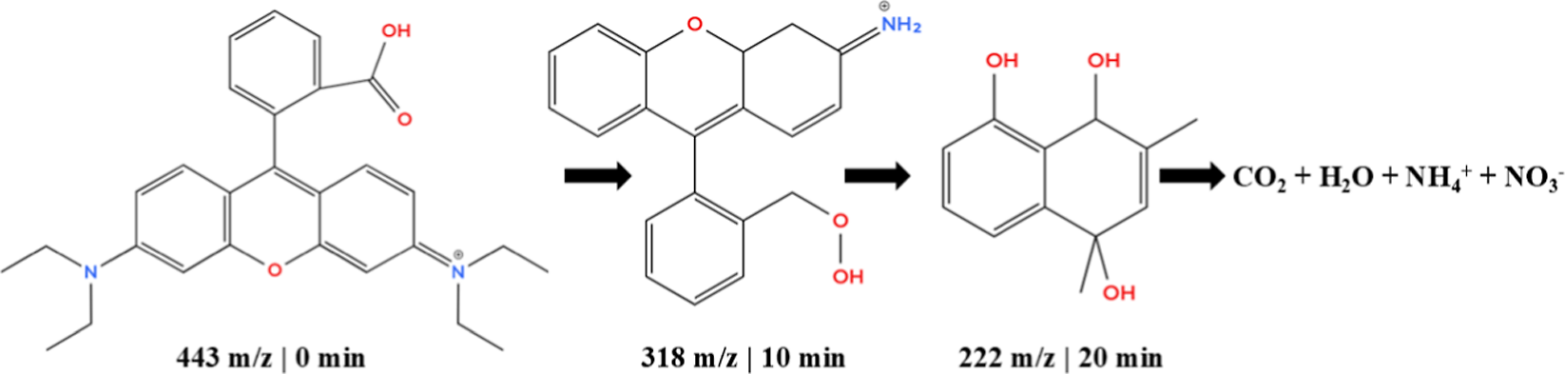
Degradation mechanism predicted by the KNN model under the conditions of this study.

In the next step (*m*/*z* 222, 20 min), the opening of the xanthene ring and the formation of a compound like dihydroxynaphthalene are observed.^60^ This intermediate is characteristic of advanced degradation processes of xanthene dyes in which the breakage of the central ring results in simpler aromatic structures that are highly prone to subsequent oxidation. The predominant presence of hydroxyl groups in these intermediates indicates the action of highly oxidizing species, such as ^•^OH, which favors both hydroxylation and successive fragmentations.^61^

As the oxidation process progresses, the remaining aromatic rings undergo further openings, which shortens the aromatic chain and progressively converts them into small organic molecules, such as low molecular weight carboxylic acids.^62^ Mineralization, which results in the formation of CO_2_, H_2_O, and inorganic ions such as NH_4_^+^ and NO_3_^–^, represents the final stage. The presence of these ions indicates that, at the end of the process,^63^ both the nitrogen and the remaining carbon skeleton have been fully oxidized.

Thus, the sequence predicts a multistep degradation process, which includes (i) dealkylation, (ii) chromophore destructuring, (iii) aromatic ring opening, and (iv) final mineralization. This behavior is aligned with the photodegradation pathways observed in xanthene dyes and provides robust evidence of the effectiveness of the oxidative process used.

### 2.9 Ecotoxicity Tests

Figure 11 presents the ecotoxic results for SiO_2_, LiNbO_3_, and SiO_2_/LiNbO_3_. Where it is possible to observe that SiO_2_ nanoparticles were toxic to brine shrimp, presenting a lethal concentration (LC_50_) of 45 μg mL^–1^, while LiNbO_3_ nanoparticles were nontoxic, and the combination of the two materials (SiO_2_/LiNbO_3_) did not show significant toxicity, indicating greater biocompatibility. In the literature, SiO_2_ presented an LC_50_ ≈ 23 μg mL^–1^ after 2 days of exposure,^64^ indicating that the SiO_2_ synthesized in the present work was less toxic to brine shrimp. *p-value: 0.05 and ** *p*-value: 0.005.

**Figure 11:**
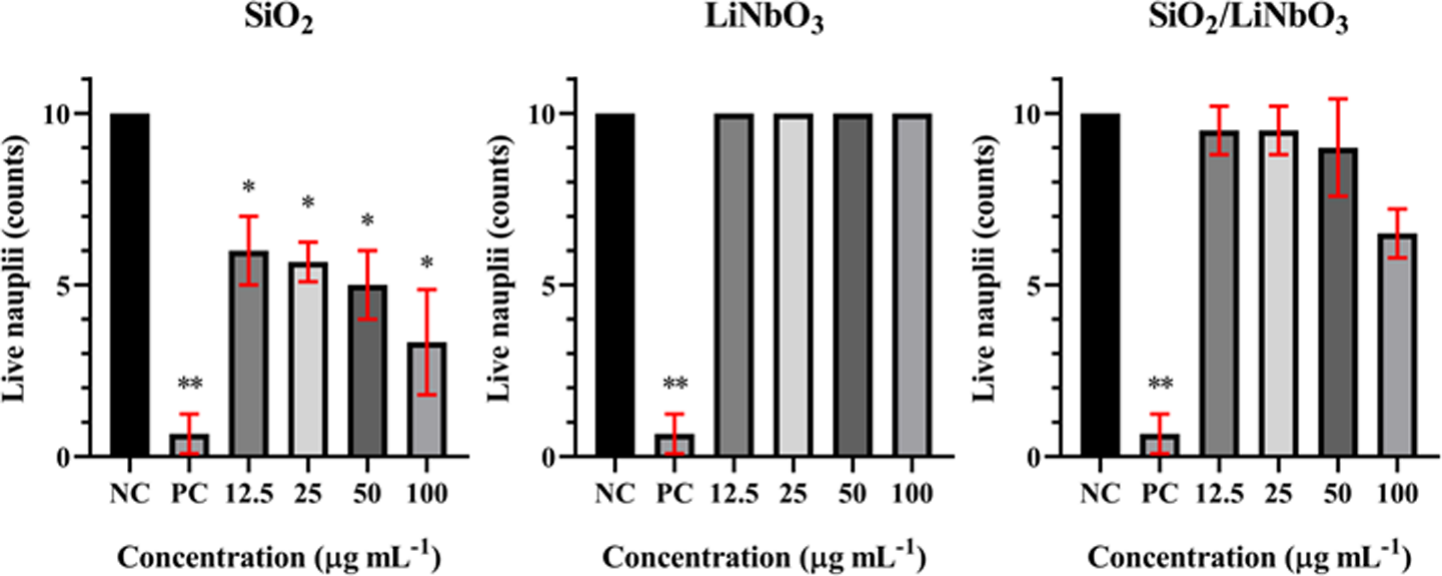
Ecotoxicity assessment of different nanoparticles in *Artemia salina* over 48 h.

### 2.10 Comparison with the Literature

The results shown in Table 5 allow for a comparative evaluation of the performance of the SiO_2_/LiNbO_3_ system within the context of photocatalytic ozonation research documented in the literature. It is noted that the catalysts used in the various studies encompass different classes of materials, such as classical semiconductors, doped catalysts, composites, and alternative materials. The SiO_2_/LiNbO_3_ system is classified as a hybrid material because it combines an inert support (SiO_2_) with a semiconductor (LiNbO_3_). This characteristic differentiates it from systems that are based only on TiO_2_ or ZnO. This variety of catalysts suggests that there is no single predominant catalytic strategy, allowing for high efficiencies to be achieved through various structural and electronic modification techniques. Regarding catalyst concentration, the values presented vary considerably, ranging from 0.1 to 8 g L^–1^. The concentration used in this study (0.2 g L^–1^) is close to the lower limit of this distribution and is lower than those of most conventional systems, especially those based on TiO_2_. From a statistical point of view, this result is significant, as it indicates that high photocatalytic efficiency can be achieved even with a reduced catalytic load, suggesting good intrinsic activity of the synthesized material. In comparison, lower concentrations offer operational advantages, such as reduced material costs, decreased dispersion problems, and possible reduction of diffusional limitations. Regarding the initial pollutant concentration, studies indicate consistent values, mostly around 10 mg L^–1^, except for some specific cases. This study used a concentration compatible with this range (10 mg L^–1^), allowing for a direct comparison between the systems and reducing distortions related to the initial organic load. Statistically, the similarity of the values indicates that variations in photodegradation efficiency are mainly linked to catalytic properties and operating conditions and not to the intensity of the analytical challenge.

**Table 5:** Comparison with Other Photocatalytic Ozonation Studies in the Literature

catalyst	concentration (g L^–1^)	pollutant	concentration (mg L^–1^)	O_3_ (mg h^–1^)	time (min)	photodegradation (%)
ZnO/MMT^65^	0.1	sulfamethoxazole	20	10,720	30	≈94.9
XC/ZnO-Cu_*x*_O^66^	0.1	salicylic acid	10	1,000	150	100
N-TiO_2_ ^67^	1	carbamazepine	10	1,008	60	99.9
fish scales/V^68^	8	malachite green	18	500	30	63.8
TiO_2_ ^69^	1	ciprofloxacin	10	340	15	98.5
SiO_2_/LiNbO_3_^this study^	0.2	RhB	10	375	40	97.2

The ozone supply rate shows greater variability among the studies, ranging from approximately 340 to 10,720 mg h^–1^. The value used in this study (375 mg h^–1^) is among the lowest in the distribution, almost at the lower limit observed. This point is especially relevant because it demonstrates that a high degradation efficiency was achieved even with a reduced oxidant dosage. Considering that ozone is an energy-intensive input, achieving high removal percentages with low feed rates indicates a more efficient use of the oxidant, possibly linked to the strengthening of the radical mechanisms caused by photocatalysis. The reaction time reported in the research also varies, ranging from 15 to 150 min. The time spent in this study (40 min) falls within the average range of the distribution. Statistically, this result can be interpreted as favorable kinetics since the system achieves high degradation in moderate times, shorter than those observed in some studies that required considerably longer periods to obtain comparable removal. In summary, the statistical analysis of the experimental variables indicates that this study achieves a high degradation efficiency under mild operating conditions, characterized by a low catalytic load, a reduced ozone rate, and a moderate reaction time. These results indicate that the SiO_2_/LiNbO_3_ system has promising performance, combining high photocatalytic activity with potential operational and economic benefits, which is important for practical applications in wastewater treatment processes.

## 3 Conclusion

The study showed that it was successful in producing a sustainable and highly effective nanocatalyst for the degradation of emerging pollutants. The synthesis of the material, which combines SiO_2_ from agro-industrial waste with lithium niobate LiNbO_3_ biosynthesized from plant extract, proves the viability of an economical, sustainable technology in accordance with the principles of circular economy. The results achieved in the research are significantly positive. Regarding catalytic efficiency, the SiO_2_/LiNbO_3_-supported nanocatalyst achieved a photodegradation rate of RhB dye greater than 97% in less than 40 min, demonstrating the high effectiveness of the photocatalytic ozonation process. This result becomes even more relevant when considering the optimized performance of the system, which showed superior synergy between the catalyst and O_3_ compared with the data available in the literature. This synergy enabled the use of lower catalyst concentrations and reduced the level of the O_3_ fluxes, leading to a decrease in the process’s energy demand. Furthermore, the study of the degradation mechanism revealed the predominant presence of the O_2_^•‑^ radicals and h^+^ holes. Ecotoxicity tests confirmed the environmental safety of the material, showing that the synthesized nanomaterials have low/no toxicity to *Artemia salina*. In conclusion, the use of machine learning to predict degradation intermediates was successful. The KNN model stood out by presenting the best performance (*R* ^2^ = 0.952 and the lowest MAE), confirming the effectiveness of machine learning in interpreting and predicting the formation of complex molecular byproducts. In summary, the study proposes a solid and sustainable solution for the treatment of effluents containing emerging pollutants using nonvalue-added waste and promoting a circular economy.

## 4 Materials and Methods

### 4.1 Reagents

All reagents used were of analytical grade: hydrochloric acid (HCl) (37%), ethyl alcohol (CH_3_CH_2_OH) (≥95%), sodium carbonate (Na_2_CO_3_) (≥99%), potassium chloride (KCl) (≥99%), sodium chloride (NaCl) (≥99%), calcium chloride dihydrate (CaCl_2_.2H_2_O) (≥99%), magnesium chloride hexahydrate (MgCl_2_.6H_2_O) (≥99%), sodium hydroxide (NaOH) (≥99%), copper sulfate (CuSO_4_) (≥99%), ammonium hydroxide (NH_4_OH) (≥99%) isopropyl alcohol (C_3_H_8_O) (≥99%), potassium dichromate (K_2_Cr_2_O) (≥99%), ascorbic acid (C_6_H_8_O_6_) (≥99%), ethylenediaminetetraacetic acid (EDTA) (C_10_H_16_N_2_O_8_) (≥99%), and sodium sulfate (Na_2_SO_4_) (≥99%) were obtained from the Synth brand. Lithium chloride (LiCl) (≥99%), sodium thiosulfate heptahydrate (Na_2_S_2_O_3_.5H_2_O) (≥99%), and niobium chloride (V) (NbCl_5_) (≥99%) were obtained from Sigma-Aldrich.

### 4.2 Synthesis

#### 4.2.1 SiO_2_

SiO_2_, used as a matrix for this work, was synthesized using 10 g of rice husk added to a 200 mL HCl solution (2 mol L^–1^) under magnetic stirring at 60 °C and 150 rpm for 1 h. The product was then washed with distilled water until pH 7 and filtered under vacuum. Afterward, the material was calcined at 600 °C with heating at 30 °C min^–1^ for 4 h to remove impurities from the husk composition^70^ as shown in Scheme 1a.

**Scheme 1:**
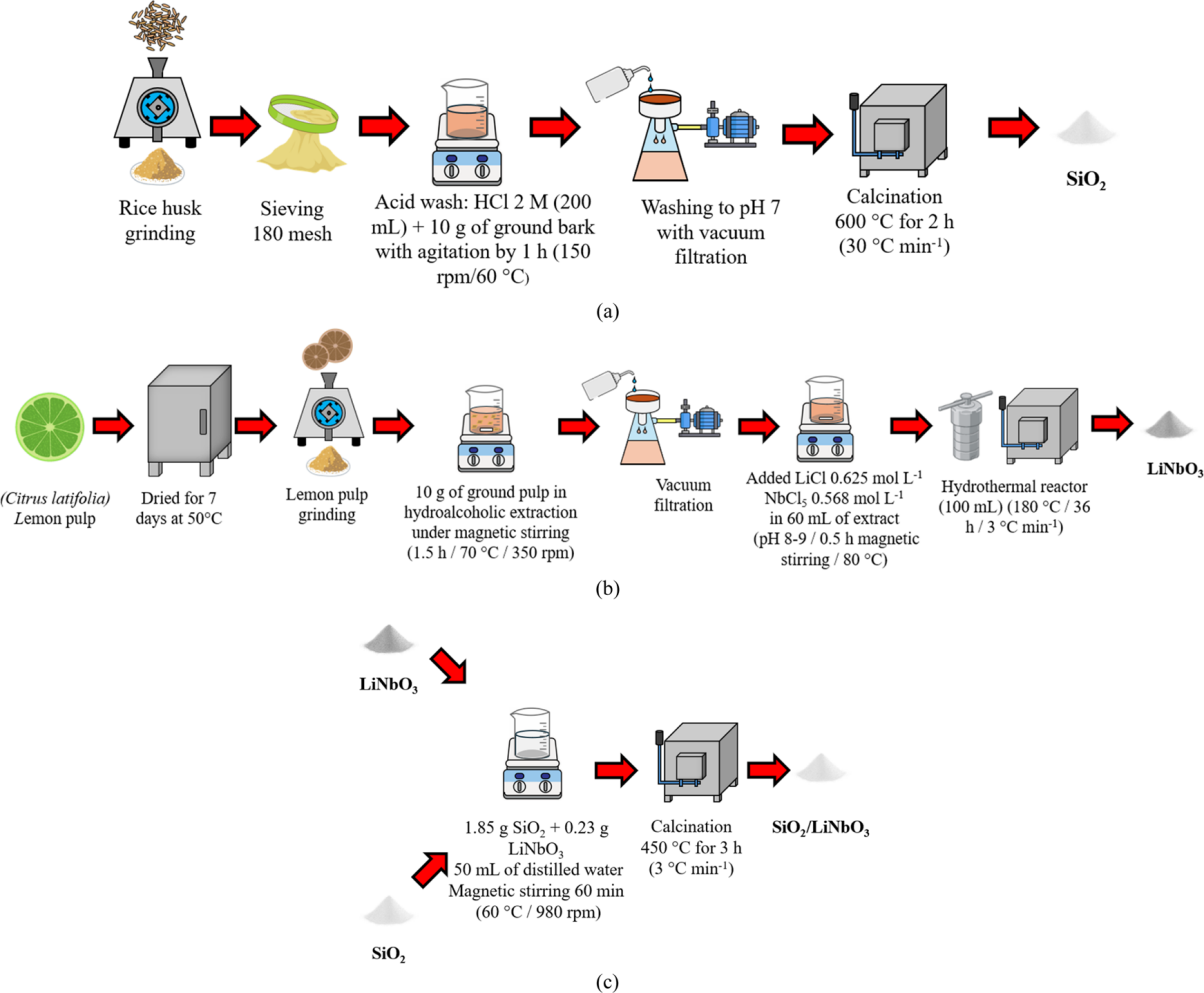
Synthesis Process: (a) SiO_2_, (b) LiNbO_3_, and (c) SiO_2_/LiNbO_3_

#### 4.2.2 LiNbO_3_

The plant extract was prepared from *C. latifolia* peels acquired from a local store (Santa Maria, RS, Brazil −29° 41′ 29″ South, 53° 48′ 3″ West) that were dried for 7 days at 50 °C, then ground in a knife mill, where 10 g of the ground peel was then placed in a hydroalcoholic solution containing 80 mL of distilled water and 20 mL of ethanol, where it remained under magnetic stirring for 1.5 h at 70 °C. For the synthesis of green nanoparticles, 60 mL of previously prepared plant extract with 0.625 mol L^–1^ of LiCl and 0.568 mol L^–1^ of NbCl_5_ was weighed at a 1.1:1 Li/Nb ratio. The pH of the solution was adjusted between 8 and 9 with NH_4_OH, and the solution was kept under magnetic stirring for 30 min at 80 °C. The chosen route was hydrothermal, using a stainless-steel autoclave reactor with 100 mL of polytetrafluoroethylene (PTFE). The material was conditioned and heated in a muffle furnace at 180 °C for 36 h at a rate of 3 °C min^–1^. Finally, the material was vacuum-filtered, washed, and then returned to the muffle furnace for stabilization before calcination at 450 °C for 4 h with a heating rate of 3 °C min^–1^. The synthesis process of LiNbO_3_ nanoparticles^71,72^ is shown in Scheme 1b.

The formation of nanoparticles using a plant extract in an autoclave reactor occurs through a unified process in which the metabolic compounds of the extract act simultaneously as reducing, complexing, and stabilizing agents. First, the plant extract provides bioactive compounds that can interact with metallic or inorganic precursors, facilitating complexation and, in many cases, the partial chemical reduction of the species present in the solution. Under high-temperature and high-pressure hydrothermal conditions, the processes of hydrolysis and structural reorganization intensify, facilitating the nucleation and subsequent growth of the nanoparticles. At this stage, the organic molecules in the extract adsorb onto the surface of the forming particles, decreasing surface energy, restricting uncontrolled growth, and preventing agglomeration, which help control the size and morphology. At the end of the process, the particles are separated and, if necessary, undergo heat treatment to eliminate organic residues and improve crystalline, generating materials produced by a more environmentally sustainable method.^73^

#### 4.2.3 SiO_2_/LiNbO_3_

For the synthesis of the supported heterogeneous nanocatalyst (SiO_2_/LiNbO_3_), the impregnation technique was used as adapted from the literature. Thus, the photoactive phase was LiNbO_3_, while SiO_2_ was the catalytic support. Thus, the proportion of 5% mol mol^–1^ (SiO_2_/LiNbO_3_) was used, followed by magnetic stirring for 60 min (70 °C/980 rpm) and calcination (450 °C, 3 h), with a heating rate of 30 °C min^–1^ ^74^ as shown in Scheme 1c.

### 4.3 Characterizations

SiO_2_, LiNbO_3_, and SiO_2_/LiNbO_3_ were characterized by Attenuated Total Reflection Fourier Transform Infrared (ATR-FTIR),^75^ Scanning Electron Microscopy Energy-Dispersive X-ray Spectroscopy (SEM-EDX).^76^ The specific areas (*S*_BET_), pore volume (*V*_p_), and pore diameter (*D*_p_)^77^ were determined by Diffuse Reflectance Spectroscopy (DRS),^78^ X-ray diffraction (XRD),^79^ and Zero Point of Charge (pH_ZCP_).^80^ More information about characterization is provided in the Supporting Information.

### 4.4 Central Composite Rotatable Design

For statistical analysis, Statistica 10 software (version 10) was used to determine the ideal conditions for pollutant and catalyst concentration, employing the CCRD methodology and response surface analysis. Thus, for the CCRD, 2 repetitions (2^2^) were used, obtaining a set of 10 experiments. For the execution of the CCRD, the independent variables and the minimum and maximum limits for RhB (5.86–34.14 mg L^–1^) and SiO_2_/LiNbO_3_ (Cat) (0.14–0.56 g L^–1^) were fixed^81^ as shown in Table 6. The SHAP (SHapley Additive exPlanations) analysis was performed in Python version 3.11.12 to interpret complex predictive models, assigning to each limit an individual contribution to the prediction of a model. The input data provided were reaction time, O_3_ mass flow rate, and Cat and RhB concentration, while the reaction rate was used as the output.^82^

**Table 6:** Limits of the Parameters Evaluated for the RhB Photodegradation by the CCRD 2^2^

	level				
independent variables	–1.41	–1	0	1	1.41
SiO_2_/LiNbO_3_ (g L^–1^)	0.14	0.20	0.35	0.50	0.56
Rh6G (mg L^–1^)	5.86	10	20	30	34.14

### 4.5 Photocatalytic Ozonation Assays

Photocatalysis tests were performed in an isolated box protected from the external radiation. A 100 mL volume of effluent was used in a 600 mL beaker with the natural pH of the solution (pH ≈ 6). An O_3_ generator (OzonAr, model Home O_3_) with a mass flow rate of 375 mg h^–1^ of O_3_ was used in the research.^83^ A 90 W 50/60 Hz 6500 K visible light (VIS) fluorescent lamp (Taschibra) with magnetic stirring at 900 rpm was used. The adsorption process was allowed to proceed for 15 min. After the lamp was activated, approximately 1 mL was collected at 0, 5, 10, 15, 20, 25, 30, 40, 50, and 60 min. These samples were centrifuged at 4500 rpm for 5 min to decant the catalyst and then analyzed by using a UV–vis spectrophotometer with a quartz cuvette at a wavelength of 556 nm.^84^ Residual O_3_ was neutralized with a solution of Na_2_S_2_O_3_ (0.1 M).

### 4.6 Kinetic Studies

The Langmuir–Hinshelwood (L–H) model is represented in eq 1 in exponential form, and eq 2 was used to determine the half-life (*t*_1/2_) (min) pseudo-first-order L–H reaction.^85^

1 $$C={C_{0}}^{*}e^{-k_{1}*t}$$

2 $$t_{1/2}=\frac{\ln\left(2\right)}{k_{1}}$$

where *C* is the concentration at time *t* and *C*_0_ is the initial concentration (mg L^–1^), *k*_1_ is the reaction rate (min^–1^), and *t* is time (min). For the kinetic study, the concentration of RhB was varied at 2.5, 5, 10, 30, and 50 mg L^–1^ by using the best catalyst concentration available.

### 4.7 Comparative Evaluation of Catalytic Systems

For the evaluation of catalytic systems alone and in combination, the ideal point obtained in the CCRD was used with 10 mg L^–1^ RhB and a flow rate of 375 mg h^–1^ of O_3_. Systems with only the O_3_, the O_3_ + separate photoactive phase (0.021 g L^–1^ of LiNbO_3_), the O_3_ + separate support phase (0.179 g L^–1^ SiO_2_), and the O_3_ + nanocatalyst supported at the ideal point (0.2 g L^–1^ SiO_2_/LiNbO_3_) were evaluated. The tests were performed under the same temperature and time conditions as the CCRD.

### 4.8 Thermodynamic Study

To better understand the photodegradation mechanism, a thermodynamic study was conducted at temperatures of 298.15, 308.15, and 318.15 K, using eqs 3–5 and the transition state (Eyring equation) and Arrhenius theories.^86,87^

3 $$\ln\left(\frac{k_{1}}{T}\right)=\ln\left(\frac{K_{B}}{h}\right)+\frac{\Delta^{‡}S^{{}^{\circ}}}{R}-\frac{\Delta^{‡}H^{{}^{\circ}}}{RT}$$

4 $$\Delta^{‡}G^{{}^{\circ}}=\Delta^{‡}H^{{}^{\circ}}-T\Delta^{‡}S^{{}^{\circ}}$$

5 $$k_{1}=A\exp^{\left(-A_{e}/RT\right)}$$

where *T* is the temperature (K), *K*_B_ is the Boltzmann constant (1.3805 × 10^–23^ J K^–1^), *h* is the Planck constant (6.6261 × 10^–34^ J s^–1^), the change in the standard enthalpy (Δ^‡^ *H* ^0^) (kJ mol^–1^) and entropy of activation (Δ^‡^ *S* ^0^) (kJ (mol K)^−1^) are brought together by the change in the standard Gibbs free energy (Δ^‡^ *G* ^0^) (kJ mol^–1^), R is the gas constant (0.008314 (kJ (mol K)^−1^)), A is the Arrhenius constant (dimensionless), and *A*_*e*_ is the activation energy of the reaction (kJ mol^–1^).

### 4.9 Effect of Scavengers on Heterogeneous Photocatalysis

To investigate the mechanism of the catalytic ozonation process, the presence of radicals (^•^OH and O_2_^–•^), valence holes (h^+^), and electrons (e^–^) was evaluated using 1 mmol of isopropyl alcohol (IA) (scavenger ^•^OH), potassium dichromate (PD) (scavenger e^–^), ascorbic acid (AA) (scavenger O_2_^–•^), and ethylenediaminetetraacetic acid (EDTA) (scavenger h^+^) for 100 mL of the solution containing the effluent.^88^

### 4.10 Reuse Tests

Reuse tests were performed under the best conditions obtained with 10 mg L^–1^ RhB and 0.2 g L^–1^ of nanocatalyst. After the photocatalysis process, the catalyst was collected and dried at 50 °C for reuse.^89^

### 4.11 Machine Learning

For the ML study, a database was created using research articles available on Google Scholar using the keywords rhodamine B AND photodegradation AND *m*/*z* AND LC-MS OR GC-MS. The table containing data such as band gap energy (*E*_g_ (eV)), time (min), peak intensity (%), dye shrinkage (mg L^–1^), and *S*_BET_ (m^2^ g^–1^) was used as input data, and the intermediate values in the mass-to-weight ratio (*m*/*z*) were used as output. In this study, ML models such as random forest (RF), multilayer perceptron (MLP), k-nearest neighbors (KNN), gradient boosting regressor (GBR), and extremely randomized trees (ERT) were used. Further information on the methodology used is available in Supporting Information Table S1.

### 4.12 Statistical Evaluation

For statistical analysis, eqs 6 and 7 were used to determine the coefficient of determination (*R* ^2^) and the mean absolute error (MAE), respectively,^90,91^ and the Shapley Additive exPlanations (SHAP) method was employed to assess the significance of the variables used in the CCRD.

6 $$R^{2}=1-\frac{\sum_{i=1}^{N}{\left(y_{\exp}-y_{pred}\right)}^{2}}{\sum_{i=1}^{N}{\left(y_{\exp}-{ŷ}_{pred}\right)}^{2}}$$

7 $$MAE=\frac{1}{2}\sum_{i=1}^{n}\left|y_{\exp}-{ŷ}_{pred}\right|$$

where *y*_exp_ and *y*_pred_ are the experimental and the predicted values, respectively; *ŷ*_pred_ is the predicted data associated with the response variable, and *n* is the number of data.

### 4.13 Ecotoxicity Tests

Ecotoxicity tests were performed using *Artemia salina*, which were exposed to solutions containing 12.5, 25, 50, and 100 μg mL^–1^ of SiO_2_, LiNbO_3_, and SiO_2_/LiNbO_3_ for 48 h. Afterward, the total number of live individuals was counted to evaluate the ecotoxicity of the synthesized nanomaterials.^92^ Further information on the methodology is available in the Supporting Information.

## Supplementary Material



## Notes

Characterization process, machine learning and its database, ecotoxicity assay specifications, DRS results, elemental mapping, and XRD (PDF)
