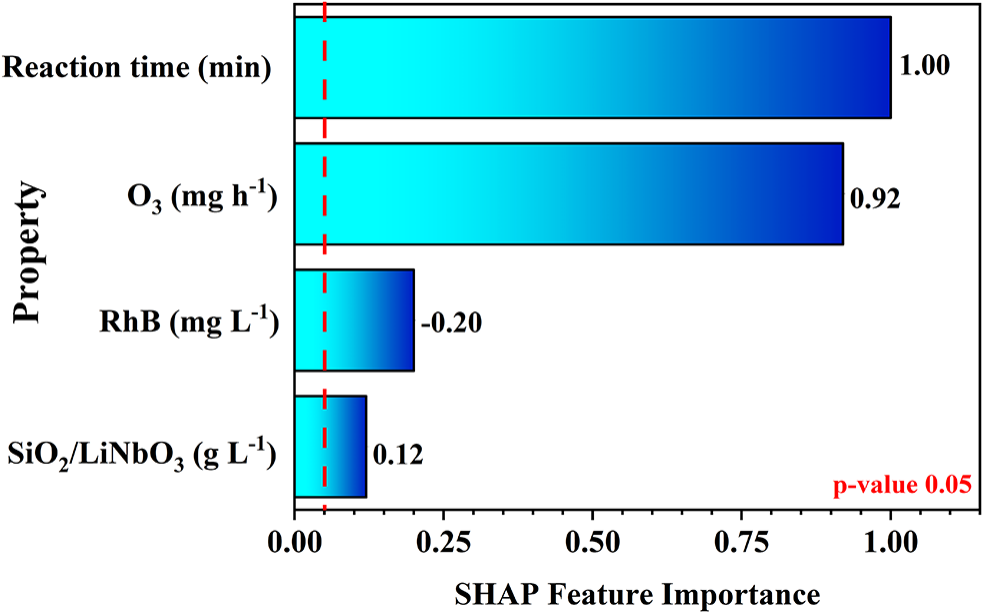# Correction to “Strategic Brazilian Minerals Applied in the Photocatalytic Ozonation of Rhodamine B Using a Green Lithium Niobate Nanocatalyst Supported on Silica: Kinetic, Thermodynamic, Mechanism, Machine Learning, and Ecotoxicity Study”

**DOI:** 10.1021/acsomega.6c03420

**Published:** 2026-06-09

**Authors:** Matheus Londero da Costa, Cristiane dos Santos, Yolice Patricia Moreno Ruiz, Giovani Pavoski, Jorge Alberto Soares Tenório, Denise Crocce Romano Espinosa, Jivago Schumacher de Oliveira

A space is missing in the surname of one author. After the change: Yolice Patricia Moreno Ruiz. This is reflected in the authorship of this Correction.

We are replacing Figure 5c, which shows the SHAP graph obtained by evaluating the photocatalytic ozonation of RhB. After the change: After adding the corrected image that was attached, a small change needs to be made to the text before the image, as previously informed in the approved email. Where the article previously stated this paragraph: "Figure 6c shows the SHAP graph obtained by evaluating the photocatalytic ozonation of RhB, where it is possible to verify that the variables with the greatest significance for the process are the reaction time (min) and the O_3_ flow rate (mg h^−1^), which have a positive effect, while the RhB concentration has a negative effect. Finally, the catalyst concentration has an intermediate positive significance in the photodegradation." Now with the new image the correct discussion is this "Figure 5c shows the SHAP graph obtained by evaluating the photocatalytic ozonation of RhB, where it is possible to verify that the variables with the greatest significance for the process are the reaction time (min) and the O_3_ flow rate (mg h^−1^), which have a positive effect, while the RhB concentration has a negative effect. Finally, the catalyst concentration has a positive significance in the photodegradation at low concentrations."