# Supplementary material for: Strategic Brazilian Minerals Applied in the Photocatalytic Ozonation of Rhodamine B Using a Green Lithium Niobate Nanocatalyst Supported on Silica: Kinetic, Thermodynamic, Mechanism, Machine Learning, and Ecotoxicity Study
Source: ACS Omega. 2026 Mar 12;11(11):18378–92. doi: 10.1021/acsomega.6c00300 (PMC13019379; doi:10.1021/acsomega.6c00300)
Supplement: Supplementary file 1 [file ao6c00300_si_001.pdf]

SUPPLEMENTARY MATERIAL

Strategic Brazilian minerals applied in the photocatalytic ozonation of rhodamine B using a green lithium niobate nanocatalyst supported on silica: Kinetic, thermodynamic, mechanism, machine learning and ecotoxicity study

Matheus Londero da Costa[a]*, Cristiane dos Santos[b], Yolice Patricia Moreno Ruiz[c,d], Giovani Pavoski[e], Jorge Alberto Soares Tenório[e], Denise Crocce Romano Espinosa[e], and Jivago Schumacher de Oliveira[a]*

[a]Applied Nanomaterials Research Group (GPNAp), Franciscan University (UFN), Santa Maria, RS, 97010-030, Brazil.

[b]Institute of Chemistry, Federal University of Rio Grande do Sul (UFRGS), Porto Alegre, RS, 90040-060, Brazil.

[c]Department of Fundamental Chemistry (DQF), Federal University of Pernambuco (UFPE), Recife, PE, 50670-901, Brazil.

[d]Strategic Technologies Center of Northeast (CETENE), Recife, PE, 50740-545, Brazil.

[e]Polytechnical School of Chemical Engineering, University of the Sao Paulo (USP), São Paulo, SP, 05508-220, Brazil.

*Email: matheus.londero@ufn.edu.br; jivago@ufn.edu.br

1. MATERIALS AND METHODS

1.1 Characterization

Attenuated Total Reflectance Fourier Transform Infrared Spectroscopy (FTIR-ATR) was used to identify the functional groups present in the samples in an IRPrestige-21 spectrophotometer (Shimadzu, Japan), in transmittance mode, after the preparation of tablets, with potassium bromide (1:3) (KBr), as support and drying agent, in the range of 4000 to 450 cm^{-1}, with a resolution of 4 cm^{-1} and 32 scans. Scanning electron microscopy (SEM) was used to evaluate the morphological properties of the synthesized samples, the average particle size was counted with the help software Image J while Energy Dispersive X-ray Spectroscopy (EDX) will allow analyzing the elemental composition of the materials, in a scanning electron microscope, model MIRA3 from TESCAN, coupled to the EDS equipment from OXFORD. X-ray diffraction was used to provide information regarding the sample crystallinity and its phases. X-ray diffraction (XRD) parameters determined include crystallization orientation, average crystallite size, crystal defects, and lattice deformities. XRD analysis was performed using a Bruker D2 Phaser instrument under the following test conditions: CuKα radiation (1.5418 Å), a theta angle range of 5° to 70° with a 0.05° increment, and an accelerating voltage and applied current of 30 kV and 30 mA, respectively. X-ray diffraction was used to provide information regarding the sample crystalline and its phases. XRD parameters determined include crystallization orientation, average crystallite size, crystal defects, and lattice deformities. XRD analysis was performed using a Bruker D2 Phaser instrument under the following test conditions: CuKα radiation (1.5406 Å), a theta angle range of 5° to 70° with a 0.05° increment, and an accelerating voltage and applied current of 30 kV and 30 mA, respectively. N_2 porosimetry was used to

determine the specific area and porosity (pore diameter and volume) of SiO_2, $LiNbO_3$, and SiO_2/ $LiNbO_3$. Initially, the samples were degassed at a pressure of 10-2 to 10-3 bar and a temperature of 120 °C for 12 h in a Gemini VII 2375 Surface Area Analyzer (Micromeritics). The specific areas (S_{BET}) were determined by the Brunauer-Emmett-Teller equation (BET method) in the range of P P_0^{-1} = 0 - 1. The pore diameter and volume values were determined by the Barrett-Johner-Halenda equation (BJH method). In order to evaluate the stability in solution and surface charge of SiO_2, $LiNbO_3$, and SiO_2/ $LiNbO_3$. The point of zero charge (pH_{ZCP}) is a technique that allows relating the pH of the solution containing the pollutant to the surface charge of the catalyst. This technique determines the range in which the adsorbent surface will be protonated, deprotonated, or neutral, through the 11-point test. For the pH_{ZCP} test, 25 mg of SiO_2, $LiNbO_3$, and SiO_2/ $LiNbO_3$ were added to 50 mL of an aqueous solution with pH values ranging from 2 to 12 in a 100 mL beaker by adding 0.1 mol L^{-1} of HCl and NaOH, under in an orbital shaker 200 rpm for 24 hours. After 24 hours, the final pH was measured using a benchtop pH meter (PHMETER Simpla pH140) calibrated with two buffer solutions (one at pH 4 and the other at pH 7). The solution containing the catalyst was then filtered and the final pH measured. Finally, a graph of the variation of the final pH versus the initial pH was created for each sample, with the pH_{ZCP} of each sample being the point where the final pH curve stabilized compared to the initial one. The band-gap energy (Eg) was determined by UV-Vis Diffuse Reflectance Spectroscopy (DRS) in a JASCOV-670 equipment ranging from 200 to 800 nm where the Kubelka-Munk function, - F(R) was used to estimate the band-gap energy.

1.2 Machine learning (ML)

The dataset (Degradation__reaction_pathway RhB.xlsx) is available on GitHub (https://github.com/MscMatheusLondero/Intermediate-generation-in-rhodamine-photodegradation).

Table S1. Models and equations used in ML.

Algorithm	Parameters	Equations
Random Forest (RF)	Tree maximum depth: None, 5, 10, 20, m<br>Number of decision trees tested: 100, 200, 300.<br>Performance measured by minimizing the mean squared error R(T), according to Eq. (S1-S3).	$R\ (T) = \frac{1}{N}\sum_{t\in T}\sum_{t\in T}(y_i - \hat{y}_i)^2$ (S1)<br>$R_i\ (T) = \frac{1}{N}\sum_{t\in T}\sum_{t\in T}(y_{pred,i} - \hat{y}_{obs,i})^2$ (S2)<br>$R_j\ (T) = \frac{1}{N}\sum_{t\in T}\sum_{t\in T}(y_{pred,j} - \hat{y}_{obs,j})^2$ (S3)<br>$R_{avg}(T) = \frac{R_i\ (T) - R_j(T)}{2}$ (S4)<br>Where: R(T) = Expected value of the sum of mean squared errors using a constant as a predictive model; N is the number of nodes used in the decision/ pattern recognition in the data (one node is equivalent to one leaf of the decision tree); y_i = observed value obtained experimentally; $\hat{y}_i$= predicted value obtained from the model; t = identifier of each node, T = mean squared error between the observed and the predicted values (response). R_i(T) and R_j(T) are the measurements of the performance of groups i and j of k-decision trees; R_{avg}(T) is the final response of the RF algorithm (average of the decisions of the n–groups made up of k–trees)
Multilayer Perceptron (MLP)	Maximum number of interactions: 5000<br>hidden layer sizes: (50,), (100,), (50,50), (100,50)	$z_j^{(l)} = \sum_{i=1}^{n_{l-1}} w_{ij}^{(l)} a_i^{(l-1)} + b_j^{(l)}$ (S5)

	activation: rectified linear unit (Relu) and hyperbolic tangent (tanh)<br>alpha: 0.0001, 0.001, 0.01.<br>Performance measured by minimizing the mean squared error *R(T)*, according to Eq. (S1)	$\hat{y} = a^{(L)}$ (S6)<br>$J = \frac{1}{N}\sum_{k=1}^{N}(y_k - \hat{y}_k)^2$ (S7)<br>Where: l = layer index; ij = indices of neurons in the previous and current layers; a = neuron activation; z = weighted sum before activation; w = weight between neurons; b = neuron bias; N = number of samples.
K-Nearest Neighbors (KNN)	Number of neighbors (*k*): 2, 3, 4, 5, 6, 7, 8, 10<br>weights: unirform, distance<br>p: 1, 2<br>Weighting function: uniform and distance-based<br>Performance measured by minimizing the mean squared error *R(T)*, according to Eq. (S1)	$R\ (T)\ = \frac{1}{N}\sum_{i=1}^{N}(y_i - \hat{y}_i)^2$ (S8)<br>where $R(T)$= expected value of the mean squared error, y_i= observed value, $\hat{y}_i$= predicted value, and *N* = number of samples. The predicted value $\hat{y}_i$is computed as the weighted average of the *k* nearest training samples according to the chosen distance metric.
Gradient Boosting Regressor (GBR)	Number of estimators: 100, 200, 300<br>Learning rate: 0.01, 0.05, 0.1, 0.3<br>Maximum depth of each weak learner (tree): 2, 3, 4, 5<br>Subsample fraction: 80% of the dataset<br>Performance measured by minimizing the mean squared error *R(T)*, according to Eq. (S1)	$y_i^{(m)} = y_i - \hat{y}_i^{(m-1)}$ (S9)<br>$\hat{y}_i^{(m)} = \hat{y}_i^{(m-1)} + \eta \cdot h_m(x_i)$ (S10)<br>where $h_m(x_i)$is the weak learner (decision tree) at iteration *m* and η is the learning rate that controls the contribution of each tree. The final prediction is the weighted sum of all trees:<br>$\hat{y} = \sum_{m=1}^{M}\eta \cdot h_m(x)$ (S11)
Extremely Randomized Trees (ERT)	Number of estimators: 100, 200, 300<br>Minimum samples split: None, 5, 10, 20<br>Maximum depth of each weak learner (tree): 2, 3, 4, 5<br>Performance measured by minimizing the mean squared error *R(T)*, according to Eq. (S1)	$\hat{y}(x) = \frac{1}{M}\sum_{M=1}^{M}h_m(x)$ (S12)

The test size 0.1 with a random state of 42 was used.

1.2 ecotoxicity tests

To evaluate the ecotoxicity tests on *Artemia salinas*, 1 L of distilled water was used to which were added: 23 g of NaCl, 11 g of $MgCl_2$ x $6H_2O$, 4 g of Na_2SO_4, 1.3 g of $CaCl_2$ x $2H_2O$ and 0.7 g of KCl, the pH of the solution was adjusted to 9 with a Na_2CO_3 solution to simulate the ecosystem and provide minerals for *Artemia salinas*.

For cyst hatching, a 1 L beaker was used, to which 500 mL of artificial seawater (previously prepared) was added, protected with aluminum foil, leaving only a small opening on the upper surface, illuminated by an 18 W lamp at a distance of approximately 30 cm. Thus, the water was aerated for 15 minutes and then 10 mg of *Artemia salina* cysts were added. Therefore, aeration was maintained throughout the hatching process with a commercial aquarium pump for 48 hours with a flow rate of 3.5 (L min^{-1}), together with controlled ambient temperature of 28 ± 2°C, salinity of 32 $\mu g.mL^{-1}$ and pH 9.

For the determination of acute toxicity and lethal concentration (LC_{50}), a negative control composed of artificial seawater without any extra substance was prepared, the positive control consisting of copper sulfate ($CuSO_4$) at a concentration of 0.1 g L^{-1}, the LC_{50} was determined via interpolation as shown in Eq. (S13).

$$LC_{50} = C_1 + \left(\frac{50 - M_1}{M_2 - M_1}\right) * (C_2 - C_1) \qquad \text{(S13)}$$

Where C_1 is the concentration with mortality below 50% in μg mL^{-1}, C_2 is the concentration above 50% in μg mL^{-1}, M_1 and M_2 are the mortality in C_1 and C_2 respectively.

The tests were performed in triplicate in test tubes containing 10 mL of the test solution containing SiO_2, $LiNbO_3$, and $SiO_2/LiNbO_3$ at concentrations of 12.5; 25; 50 and 100 μg mL^{-1}, 10 nauplii were added and kept at a temperature of 28 ± 2°C for 48

hours with a photoperiod of 12 h light:12 h dark, after which the dead nauplii were counted. Tukey's variance test was used for the statistical significance analysis.

2. RESULTS

2.1 DRS

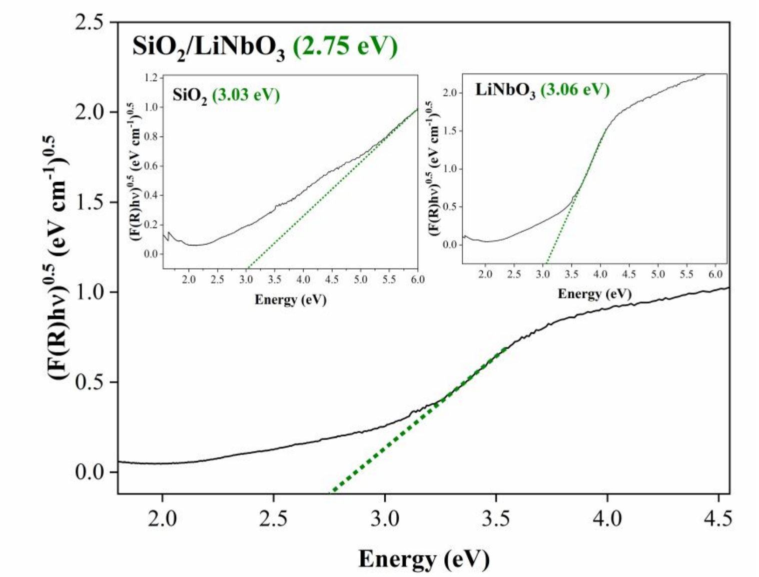

Figure S1: SiO_2, $LiNbO_3$ and $SiO_2/LiNbO_3$ DRS spectrum.

2.2 EDX

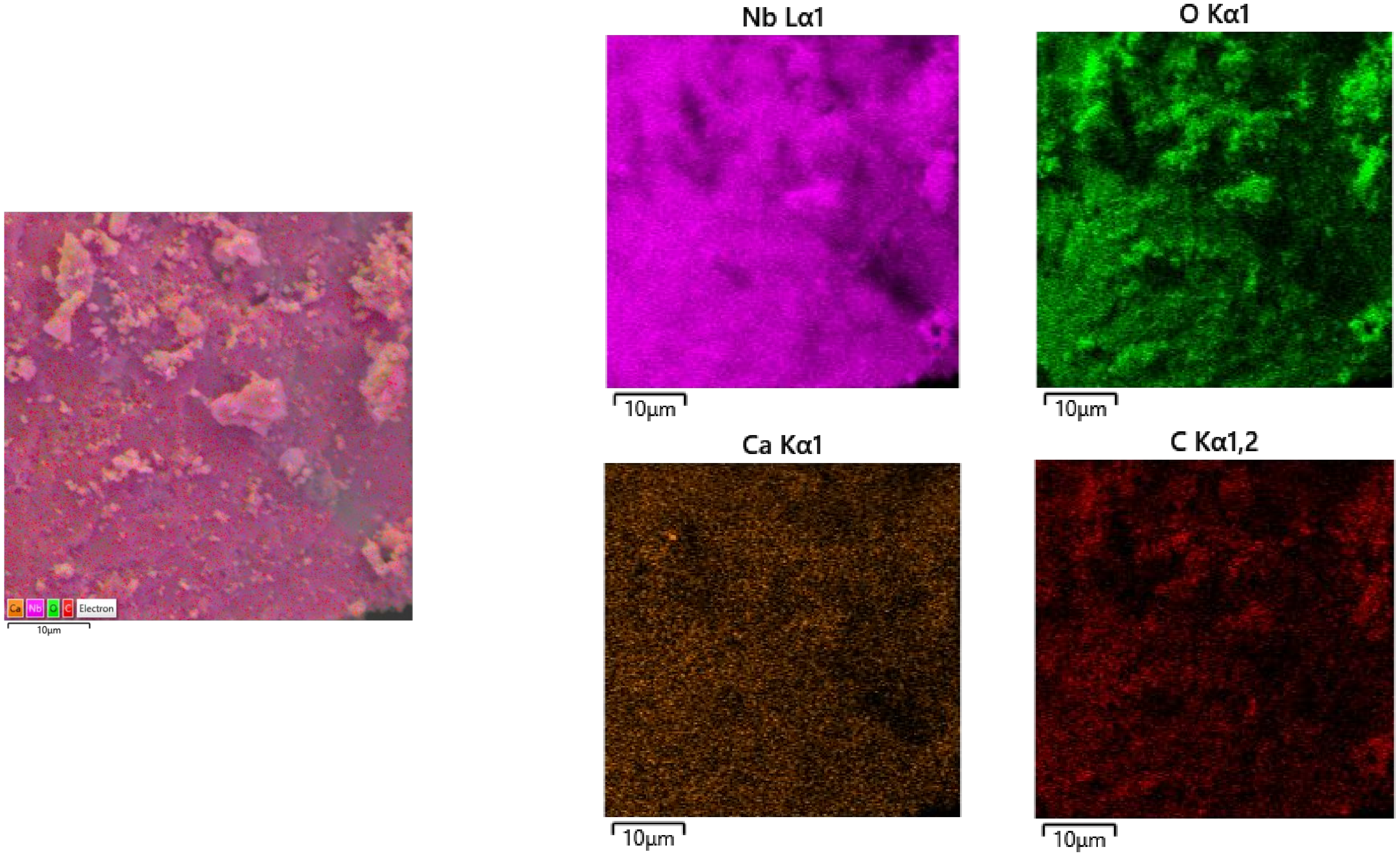

(a) $LiNbO_3$

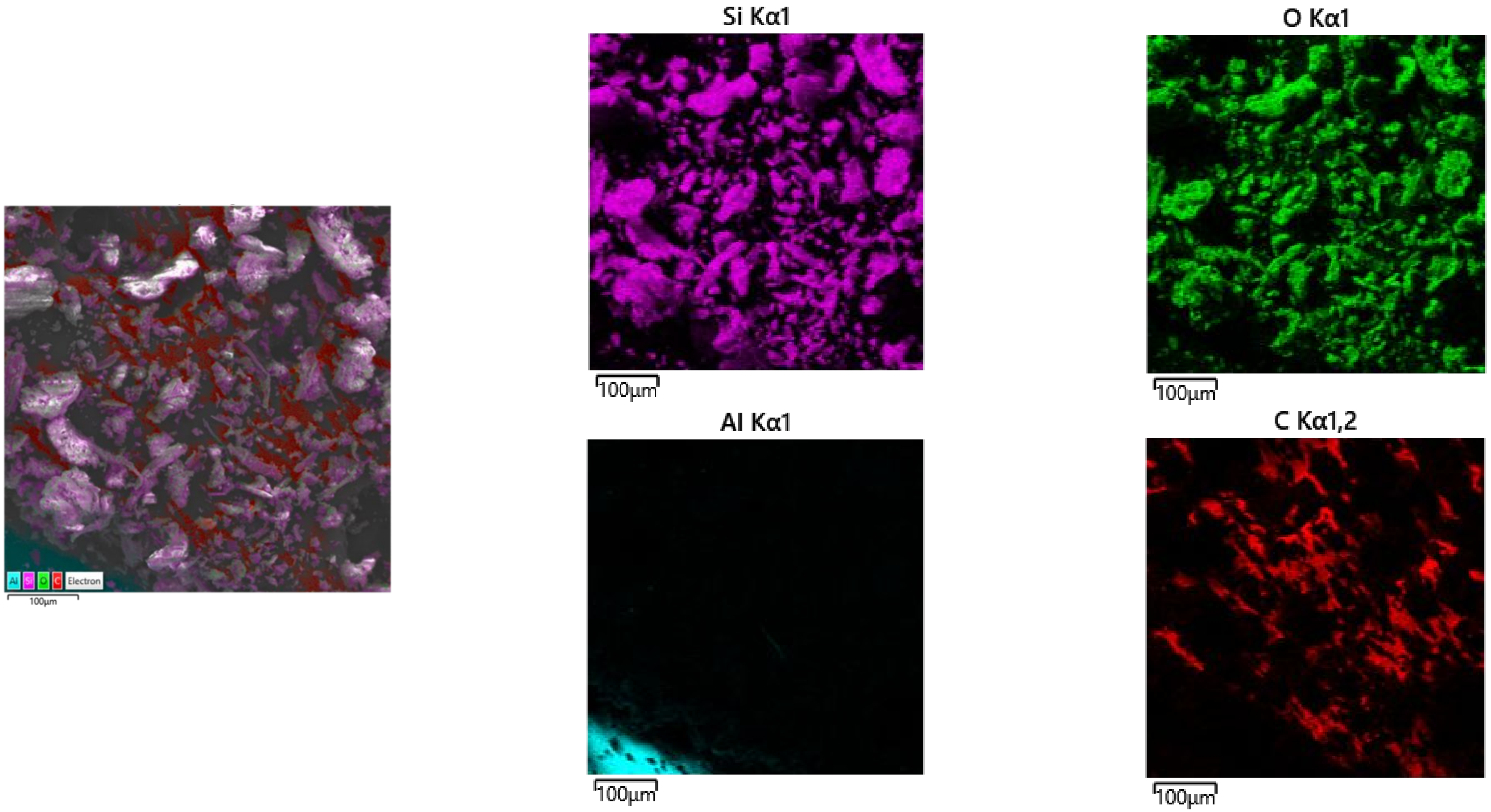

Al
Si
O
C
Electron
100μm
Si Kα1
O Kα1
Al Kα1
C Kα1,2
100μm
100μm
100μm
100μm

(b) SiO_2

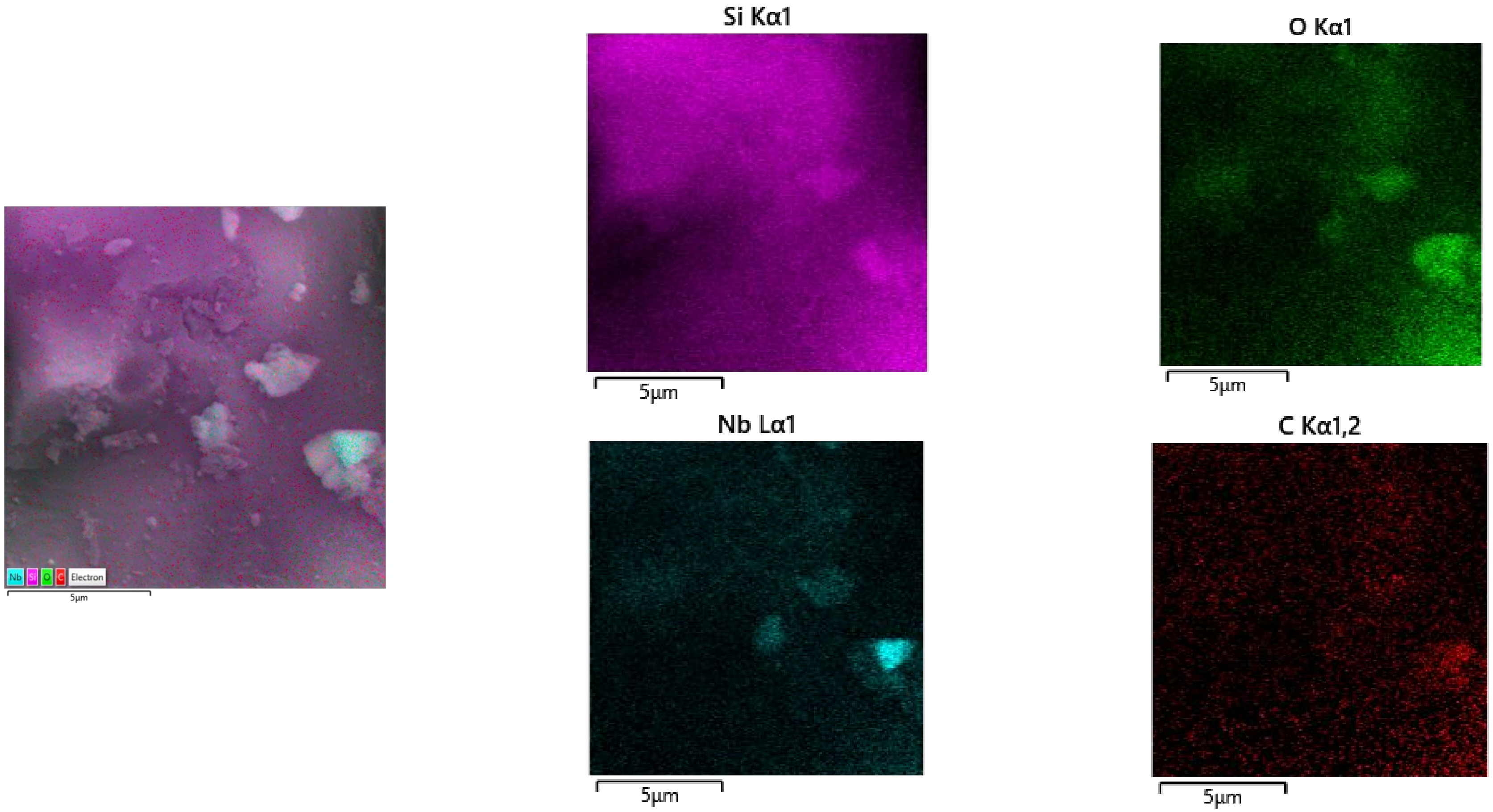

(c) $SiO_2/LiNbO_3$

Figure S2: elemental mapping of samples of (a) $LiNbO_3$, (b) SiO2 and (c) $SiO_2/LiNbO_3$.

2.3 XRD

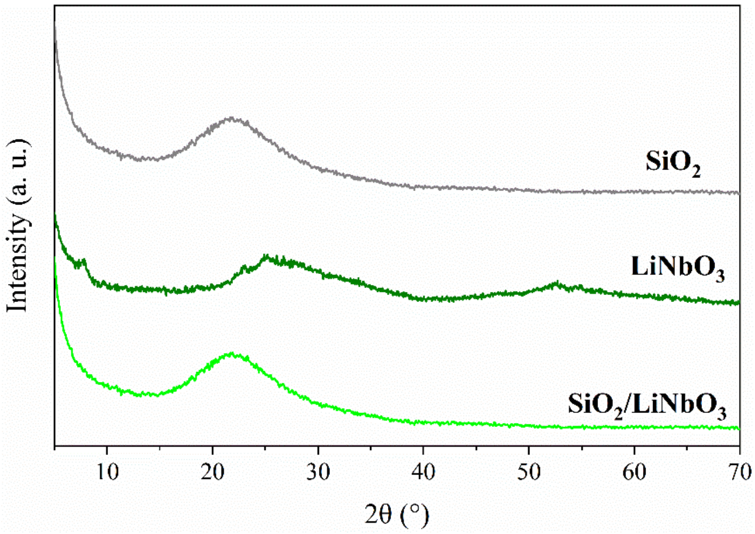

Figure S3: XRD diffraction diagram of SiO_2, $LiNbO_3$ and $SiO_2/LiNbO_3$
